# The Antidepressant Amitriptyline Upregulates ERK1/2 Signaling and Inhibits Rho-Mediated Responses Induced by Lysophosphatidic Acid in Astroglial Cells

**DOI:** 10.3390/ijms27083660

**Published:** 2026-04-20

**Authors:** Maria C. Olianas, Simona Dedoni, Pierluigi Onali

**Affiliations:** 1 ExplorePharma S.r.l., Parco Scientifico e Tecnologico della Sardegna, Edificio 5, 09010 Pula, CA, Italy; pl.onali@explorepharma.it; 2 Department of Biomedical Sciences, Division of Neuroscience and Clinical Pharmacology, University of Cagliari, 09142 Cagliari, CA, Italy; dedoni@unica.it

**Keywords:** amitriptyline, lysophosphatidic acid, ERK1/2 signaling, Rho–ROCK signaling, C6 glioma cells, rat astrocytes

## Abstract

(1) Different classes of antidepressant drugs have been shown to activate lysophosphatidic acid (LPA) receptors, but their effects on the receptor signaling stimulated by LPA have not been fully investigated. In the present study, we examined the effect of the tricyclic antidepressant amitriptyline on the LPA-induced activation of extracellular signal-regulated kinases 1 and 2 (ERK1/2) and Rho signaling in C6 glioma cells and cultured rat astrocytes. (2) LPA receptor signaling was investigated by using Western blot and microscopic immunofluorescence assays. Rho activation was determined by a pull-down assay. (3) Amitriptyline potentiated the LPA-induced activation of ERK1/2 signaling, as indicated by the more than additive increases in the phosphorylation/activation of key components of this pathway including fibroblast growth factor 1 receptor, MEK1/2, ERK1/2, Elk-1, and cyclic AMP response element binding protein (CREB). Amitriptyline also enhanced the expression of brain-derived neurotrophic factor (BDNF) elicited by LPA. In contrast, the antidepressant failed to mimic the LPA-induced activation of Rho and Rho-dependent responses, such as the reversal of astrocyte stellation, accumulation of stress fibers, and the phosphorylation of focal adhesion kinase and myosin target subunit of myosin phosphatase isoform 1. Moreover, when combined with LPA, amitriptyline curtailed Rho activation and the Rho-mediated cellular responses. (4) These results demonstrate that in astroglial cells, amitriptyline exerts a balanced action on LPA-activated receptors by enhancing the neuroprotective ERK1/2-CREB-BDNF signaling and dampening the potentially detrimental Rho–ROCK pathway, and suggest that this unique property may contribute to the antidepressant activity of the drug.

## 1. Introduction

It is currently recognized that the potentiation of monoaminergic transmission does not fully explain the therapeutic activity of antidepressant drugs and that additional molecular mechanisms are involved in their action [1]. The identification and characterization of new mechanisms are crucial steps toward the development of compounds with an improved antidepressant profile [2].

In the last decade, substantial evidence has been provided indicating that the receptors of lysophosphatidic acid (LPA), a major bioactive phospholipid, are a novel molecular target of different classes of antidepressant drugs. LPA acts through at least six G protein-coupled receptors (GPCRs), termed LPA_1–6_, which regulate the activity of the heterotrimeric G proteins G_i/o_, G_q/11_, G_12/13_ and G_s_ [3,4,5,6]. We first reported that in CHO-K1 fibroblasts the activation of endogenously expressed LPA_1_ receptors mediated the action of tricyclic and tetracyclic antidepressants on insulin-like growth factor-1 receptor transactivation, extracellular signal-regulated kinases 1 and 2 (ERK1/2) stimulation and mitogenesis [7].

Following this original observation, subsequent studies in a variety of cellular types demonstrated the involvement of LPA_1_ receptors in the effects of antidepressants, including protection from oxidative stress and induction of growth factor expression in astroglial cells [8,9], neuroprotection and suppression of TNF-α-induced apoptosis in hippocampal neurons [10,11], inhibition of p-glycoprotein transport activity in brain capillaries [12], and promotion of dermal and lung fibroblast differentiation [13].

Preclinical studies have demonstrated that constitutive deletion of LPA_1_ receptors is associated with altered neurogenesis [14], phenotypic changes resembling psychiatric diseases [15], hypersensitivity to chronic stress [16], and anxiety-like behaviors [17,18]. Meanwhile, clinical investigations have found decreased serum and cerebrospinal fluid levels of LPA and autotaxin, the major LPA-producing enzyme, in patients with major depressive disorder and schizophrenia [19,20].

More recently, by employing human embryonic kidney-293 cells individually transfected with human LPA_1_, LPA_2_ and LPA_3_ receptor cDNAs, we reported that tricyclic and tetracyclic antidepressants were able to activate the ERK1/2 pathway in all three cell systems, with different relative intrinsic activities and potencies as a function of the molecular structure and receptor subtype [21]. These results indicated that the antidepressant drugs could have a wide impact on LPA signaling by acting at all three members of the EDG family of LPA receptors. Moreover, the latter study showed that amitriptyline and mianserin, two prototypes of tricyclic and tetracyclic antidepressants, respectively, potentiated LPA-induced ERK1/2 stimulation but inhibited LPA-induced Rho activation, indicating that these drugs do not act simply as LPA receptor agonists but are also able to differentially modulate the coupling of LPA receptors to different signaling pathways. However, this finding was mainly obtained in cells overexpressing the LPA receptor subtypes and provided no information on the ability of antidepressants to differentially affect LPA-regulated cellular events occurring downstream of ERK1/2 and Rho.

To further address this issue, in the present study we investigated the effects of amitriptyline on the LPA-induced activation of ERK1/2 and Rho signaling in C6 glioma cells and rat cortical astrocytes, two cell systems in which the activation of these intracellular pathways by endogenously expressed LPA receptors is known to elicit well-characterized biochemical and morphological responses.

## 2. Results

### 2.1. Amitriptyline Potentiates LPA-Induced ERK1/2 Signaling in Astroglial Cells

**Figure 1:**
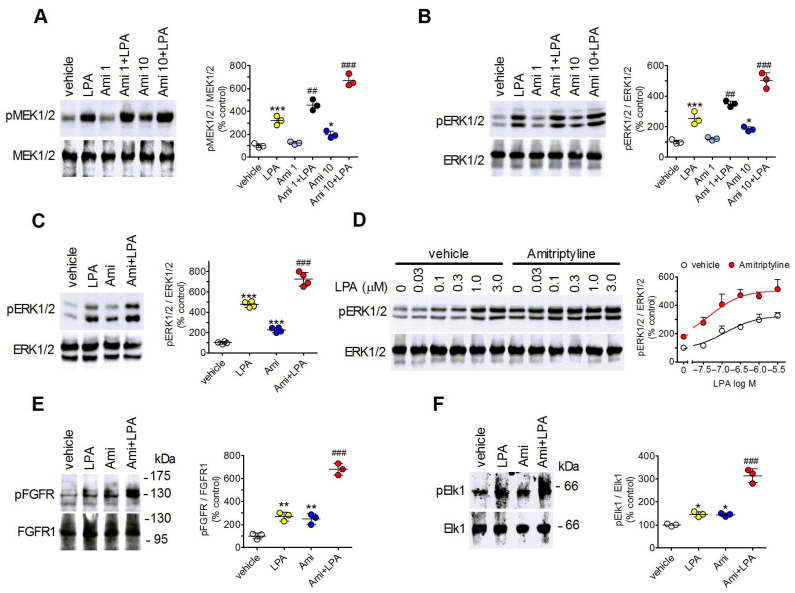
Potentiation of LPA-stimulated ERK1/2 signaling pathway by amitriptyline. (**A**,**B**) C6 glioma cells were incubated for 5 min with either vehicle, 1 µM amitriptyline (Ami 1) or 10 µM amitriptyline (Ami 10) and then exposed for 5 min to either vehicle or 0.3 µM LPA. Cell lysates were analyzed for phospho-MEK1/2 (pMEK1/2), total MEK1/2 (**A**), phospho-ERK1/2 (pERK1/2) and total ERK1/2 (**B**) immunoreactivity by Western blot. Values are the mean ± SD of three independent experiments. (**C**) Rat cortical astrocytes were incubated for 5 min with either vehicle or 10 µM amitriptyline (Ami) and then exposed for 5 min to either vehicle or 1 µM LPA. Values are the mean ± SD of four independent experiments. (**D**) C6 glioma cells grown in 2 six-well plates were incubated for 5 min with either vehicle or 10 µM amitriptyline and then exposed for 10 min to the indicated concentrations of LPA. Cell lysates were analyzed for pERK1/2 and ERK1/2. Values are expressed as percent of control (vehicle + vehicle) and are the mean ± SD of three independent experiments. (**E**) C6 glioma cells were incubated for 5 min with either vehicle or 10 µM amitriptyline and then treated for 5 min with either vehicle or 0.3 µM LPA. Cell lysates were analyzed for phospho-FGFR (pFGFR) and FGFR1 levels. Values are the mean ± SD of three independent experiments. (**F**) Rat cortical astrocytes were treated as indicated in C and cell lysates were analyzed for phospho-Elk-1 (pElk1) and total Elk-1 levels. Values are the mean ± SD of three independent experiments. The position of molecular weight standards is reported on the right side of immunoblots. * *p* < 0.05, ** *p* < 0.01, *** *p* < 0.001 vs. control (vehicle-treated cells); ^##^ *p* < 0.01, ^###^ *p* < 0.001 vs. LPA alone by Anova followed by Newman–Keuls test.

The dual-specificity kinases MEK 1 and 2 (MEK1/2) operate along the Ras–Raf signaling cascade activated by growth factors, being phosphorylated and activated by Raf, and in turn inducing the phosphorylation/activation of ERK1/2. Treatment of C6 glioma cells with LPA (0.3 µM) increased the phosphorylation state of MEK1/2 and ERK1/2 by 220 ± 40% (*p* < 0.001) and 150 ± 23% (*p* < 0.001), respectively (Figure 1A,B). Amitriptyline (1 µM) failed to affect the phosphorylation of MEK1/2 and ERK 1/2 but potentiated the LPA stimulations by 53 ± 16% (*p* < 0.05, Student’s *t* test) and 48 ± 15% (*p* < 0.05, Student’s *t* test), respectively. At 10 µM, amitriptyline increased MEK1/2 and ERK1/2 phosphorylation by 96 ± 30% (*p* < 0.05) and 83 ± 15% (*p* < 0.05), respectively. When amitriptyline was combined with LPA the net stimulations elicited by LPA were enhanced by 114 ± 26% (*p* < 0.01, Student’s *t* test) and 73 ± 12% (*p* < 0.01, Student’s *t* test), respectively (Figure 1A,B). Treatment of rat astrocytes with either LPA (1 µM) or amitriptyline (10 µM) increased phospho-ERK1/2 levels by 370 ± 29% (*p* < 0.001) and 125 ± 25% *(p* < 0.001), respectively. When astrocytes were pretreated with the antidepressant, the stimulation elicited by LPA was enhanced up to 630 ± 57%, yielding a potentiation of 36 ± 13% (*p* < 0.05, Student’s *t* test) (Figure 1C).

To characterize the facilitatory effect of amitriptyline on the LPA-induced activation of ERK1/2, concentration–response curves of LPA were performed in the absence and in the presence of amitriptyline. As shown in Figure 1D, in C6 glioma cells LPA stimulated ERK1/2 phosphorylation with an EC_50_ value of 123 ± 16 nM and an E_max_ value corresponding to a 2.3 ± 0.1-fold increase in control value. Pretreatment of cells with 10 µM amitriptyline decreased the LPA EC_50_ value to 60 ± 16 nM (*p* < 0.05, Student’s *t* test) and increased the net stimulation elicited by LPA by 43 ± 4% (*p* < 0.05, Student’s *t* test).

We have previously reported that in C6 glioma cells the activation of the LPA_1_ receptor by either LPA or antidepressants increased ERK1/2 phosphorylation through transactivation of growth factor receptors, such as the fibroblast growth factor (FGF) receptor and the platelet-derived growth factor receptor [8]. As shown in Figure 1E, exposure of C6 glioma cells to either LPA (0.3 µM) or amitriptyline (10 µM) increased the phosphorylation of the FGF receptor at the tyrosine residues 653/654, which is essential for receptor signaling [22], by 169 ± 32% (*p* < 0.01) and 148 ± 43% (*p* < 0.01), respectively. The combined treatment with amitriptyline and LPA increased the levels of FGF receptor phosphorylation by 588 ± 50%, potentiating the LPA effect by 257 ± 34% (*p* < 0.001, Student’s *t* test).

To assess whether the potentiation of LPA-induced ERK1/2 phosphorylation elicited by amitriptyline was associated with enhanced enzyme activity we examined the phosphorylation of the nuclear transcription factor Elk-1, a direct substrate of ERK 1/2. In rat astrocytes, LPA (1 µM) and amitriptyline (10 µM) stimulated Elk-1 phosphorylation at Ser383 by 45 ± 11% (*p* < 0.05) and 43 ± 7% (*p* < 0.05), respectively, whereas the combination of the two agonists produced a more than additive effect, increasing the LPA effect by 380 ± 49% (*p* < 0.001, Student’s *t* test) (Figure 1F).

### 2.2. Synergistic Interaction Between Amitriptyline and LPA in Inducing CREB Phosphorylation

**Figure 2:**
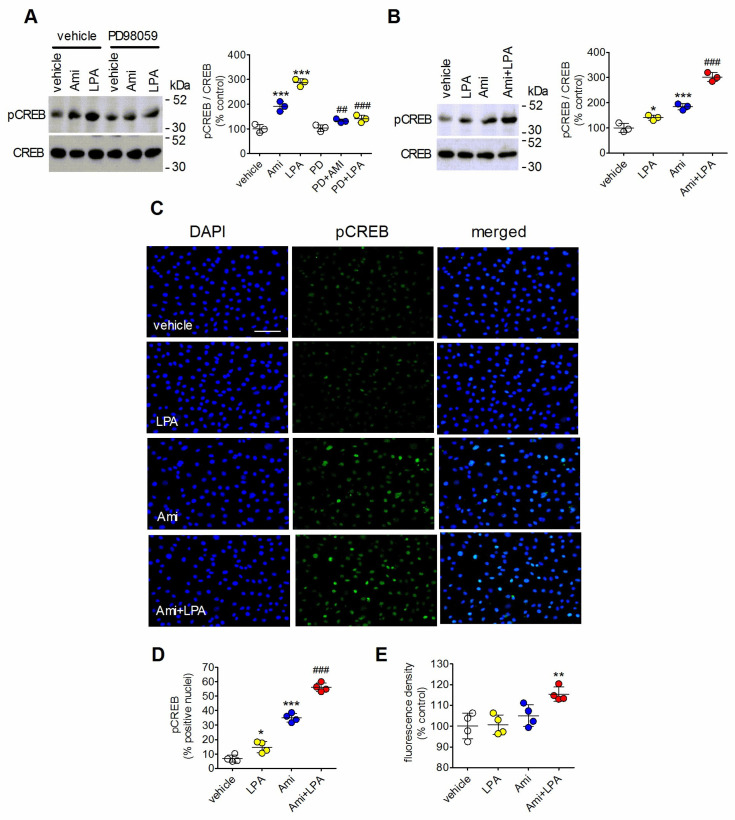
Amitriptyline enhances LPA-induced CREB phosphorylation. (**A**) C6 glioma cells were incubated for 60 min with either vehicle or 50 µM PD98059 and then treated for 30 min with either vehicle, 10 µM amitriptyline (Ami) or 10 µM LPA. Cell lysates were analyzed for phospho-CREB and total CREB levels by Western blot. Values are the mean ± SD of three independent experiments. (**B**) C6 glioma cells were incubated for 5 min with either vehicle or 10 µM amitriptyline and then treated for 30 min with either vehicle or 0.3 µM LPA. Values are the mean ± SD of three experiments. The position of molecular weight standards is indicated on the right side of immunoblots. (**C**) C6 glioma cells were treated as indicated in B and analyzed for phospho-CREB immunoreactivity (green color) by fluorescence microscopy. Nuclei were stained in blue color with DAPI. Bar = 50 µm. (**D**) The number of phospho-CREB positive nuclei are reported as percent of total nuclei. (**E**) The integrated density of phospho-CREB fluorescence in each experimental group is reported as percent of control. Values are the mean ± SD of four independent experiments. * *p* < 0.05, ** *p* < 0.01, *** *p* < 0.001 vs. control (vehicle-treated cells); ^##^ *p* < 0.01, ^###^ *p* < 0.001 vs. LPA alone by Anova followed by Newman–Keuls test.

The transcription factor cyclic AMP response element binding protein (CREB) is an additional nuclear target of ERK1/2 signaling [23]. Figure 2A shows that in C6 glioma cells the stimulation of CREB phosphorylation/activation at Ser133 [24] induced by either amitriptyline (10 µM) or LPA (10 µM) was prevented by preincubation with the MEK 1/2 inhibitor PD98059 (25 µM), indicating that both agents required ERK1/2 signaling for CREB activation. We therefore investigated whether the potentiation of LPA-induced ERK1/2 activation by amitriptyline translated into a greater stimulation of CREB.

As shown in Figure 2B, LPA (0.3 µM) and amitriptyline (10 µM) increased CREB phosphorylation by 40 ± 10% (*p* < 0.05) and 83 ± 13% (*p* < 0.001), respectively. When the two agents were combined a more than additive response was obtained (202 ± 18% increase, *p* < 0.001), with a potentiation of the LPA stimulation of 297 ± 54% (*p* < 0.001, Student’s *t* test). Quantitative analysis of phospho-CREB immunofluorescence in C6 glioma cells indicated that LPA (0.3 µM) and amitriptyline (10 µM) increased the percentage of positive nuclei by 1.1-fold (*p* < 0.05) and 4.5-fold (*p* < 0.001), respectively (Figure 2C,D). The association of amitriptyline with LPA potentiated the LPA stimulation by 3-fold (*p* < 0.001, Student’s *t* test). The resulting integrated density value of phospho-CREB immunofluorescence was significantly higher, as compared to control, only in cells treated with the combination of amitriptyline with LPA (Figure 2E).

### 2.3. Amitriptyline Upregulates LPA Induction of BDNF Expression

The gene encoding the neurotrophin brain-derived neurotrophic factor (BDNF) constitutes a major target of CREB transcriptional activity. Activated CREB directly binds to CRE elements present in the promoters of the BDNF gene and recruits the coactivator CREB binding protein to trigger BDNF transcription [25]. We investigated whether the positive interaction between amitriptyline and LPA observed in CREB activation was accompanied by a similar regulation in BDNF expression.

**Figure 3:**
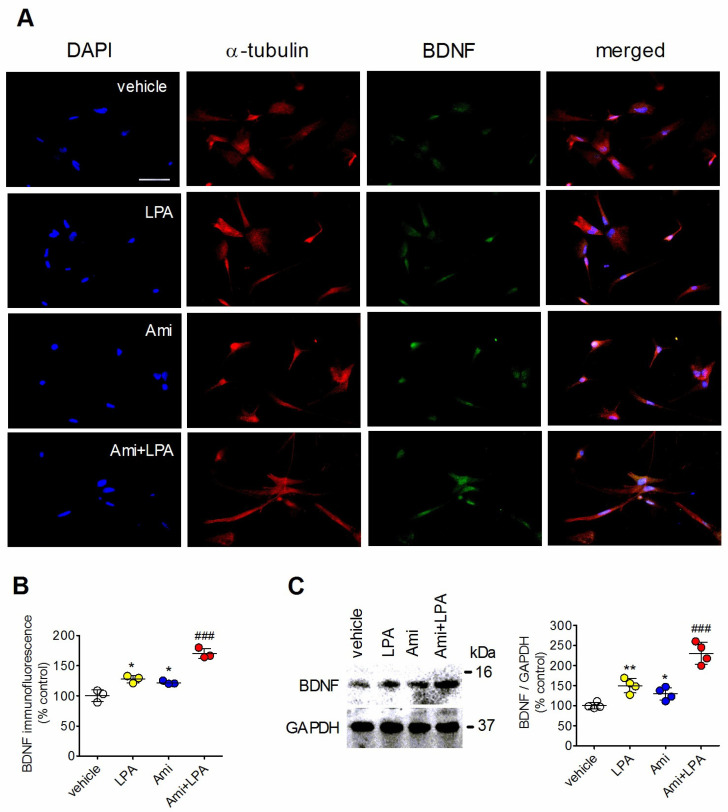
Enhancement of LPA-induced BDNF expression by amitriptyline. (**A**) Rat cortical astrocytes were incubated for 20 h in medium supplemented with 5% charcoal-stripped fetal bovine serum (St. Louis, MO, Sigma Aldrich) and treated with either vehicle, 10 µM amitriptyline (Ami), 1 µM LPA, or amitriptyline plus LPA (Ami + LPA). Cells were then analyzed for α-tubulin (red color) and BDNF (green color) immunoreactivities by fluorescence microscopy. Nuclei were stained with DAPI (blue color). Bar = 50 µm. (**B**) Values of BDNF immunofluorescence are reported as percent of control value and are the mean ± SD of three independent experiments. (**C**) Astrocytes were incubated and treated as indicated in (B) and cell lysates were analyzed for BDNF and glyceraldehyde 3-phosphate dehydrogenase (GAPDH) immunoreactivity by Western blot. The position of molecular weight standards is reported on the right side of immunoblots. Values are the mean ± SD of four independent experiments. * *p* < 0.05, ** *p* < 0.01 vs. control (vehicle treated cells); ^###^ *p* < 0.001 vs. LPA alone by Anova followed by Newman–Keuls test.

Both amitriptyline (10 µM) and LPA (1 µM) significantly enhanced the levels of BDNF protein in C6 glioma cells and rat astrocytes. The stimulatory effect of either agent was antagonized by the selective LPA_1_ receptor antagonist AM966 (100 nM), implying the involvement of this LPA receptor subtype (Supplementary Figure S1). Immunofluorescence analysis of rat astrocytes showed that the combined treatment with amitriptyline and LPA produced an increase in BDNF expression that was 45 ± 10% (*p* < 0.05, Student’s *t* test) greater than the sum of the individual effects of the two agents, indicating the occurrence of synergism (Figure 3A,B). In Western blot analysis, LPA (1 µM) and amitriptyline (10 µM) increased BDNF protein levels by 50 ± 18% (*p* < 0.05) and 29 ± 16% (*p* < 0.05), respectively (Figure 3C). The combined treatment with the two agents increased the levels of BDNF to 130.4 ± 27%, yielding a potentiation of LPA stimulation equal to 75 ± 12% (*p* < 0.05, Student’s *t* test).

### 2.4. Amitriptyline Fails to Stimulate Rho and Inhibits Rho Activation Induced by LPA

**Figure 4:**
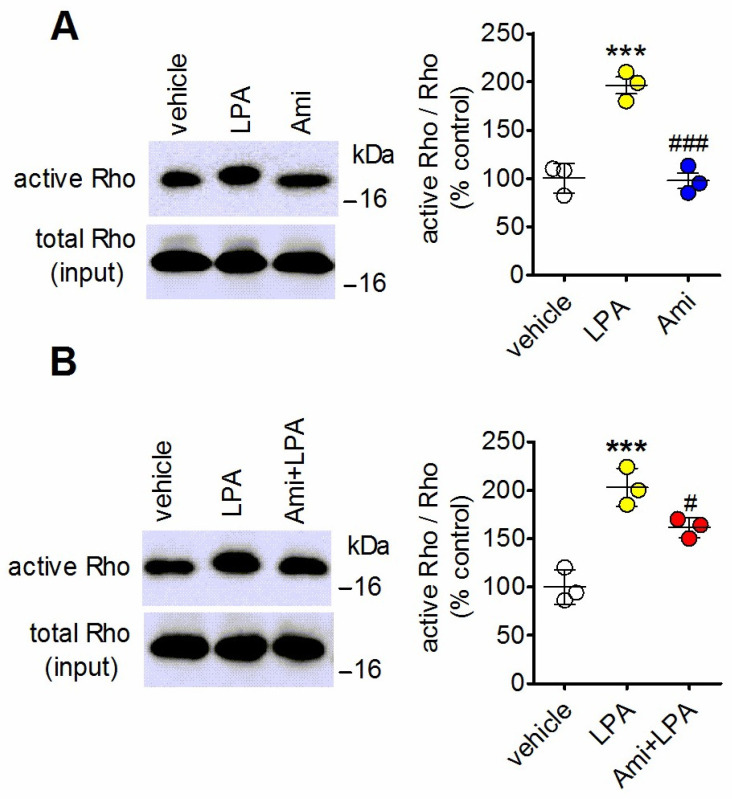
Amitriptyline curtails LPA-induced activation of Rho. (**A**) C6 glioma cells were incubated for 10 min with either vehicle, 10 µM LPA or 10 µM amitriptyline (Ami). (**B**) Cells were incubated for 5 min with either vehicle or 10 µM amitriptyline and then treated for 10 min with either vehicle or 10 µM LPA. Cell extracts were analyzed for active and total Rho by Western blot. Densitometric ratios are expressed as percent of control and are the mean ± SD of three independent experiments. *** *p* < 0.001 vs. control (vehicle-treated cells); ^#^ *p* < 0.05, ^###^ *p* < 0.001 vs. LPA alone by Anova followed by Newman–Keuls test.

Exposure of C6 glioma cells to LPA (10 µM) increased the levels of active Rho by 96 ± 15% (*p* < 0.001), whereas, under the same experimental conditions, amitriptyline (10 µM) had no significant effect (Figure 4A). In cells pretreated with the antidepressant Rho, activation induced by LPA was reduced by 40 ± 9% (*p* < 0.05, Student’s *t* test) (Figure 4B).

### 2.5. Amitriptyline Impairs LPA Reversal of Isoproterenol-Induced Morphological Changes in Astrocytes

**Figure 5:**
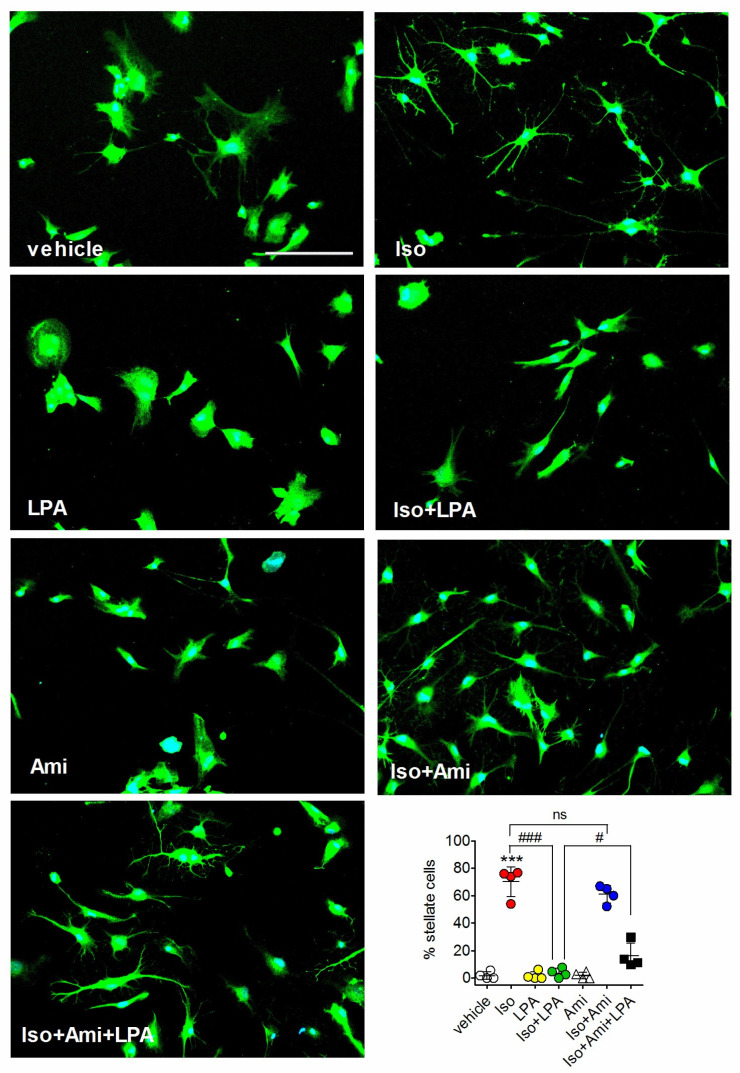
Amitriptyline fails to mimic LPA reversal of l-isoproterenol-induced astrocyte stellation and counteracts the response to the phospholipid. Rat cortical astrocytes were incubated in serum-free medium and treated for 2 h with either vehicle or 10 µM l-isoproterenol (Iso). Thereafter cells were treated with either vehicle or 1 µM LPA and the incubation was continued for 1 h. Amitriptyline (Ami) (10 µM) was added 10 min before the exposure to vehicle or LPA. Cells were then analyzed for GFAP immunoreactivity (green color) by fluorescence microscopy. Cell nuclei were stained in blue color with DAPI. Bar = 125 µm. The number of stellate cells in each experimental group is reported as percent of total cells. Values are the mean ± SD of four independent experiments. *** *p* < 0.001 vs. control (vehicle-treated cells); ^#^ *p* < 0.05, ^###^ *p* < 0.001; ns = not statistically significant, by Anova followed by Newman–Keuls test.

Treatment of astrocytes with cyclic AMP-stimulating agents or cyclic AMP analogs in serum-free conditions is known to induce profound structural changes, such as retraction of cytoplasm toward the perinuclear region, rounding of the soma, and the formation of thin processes, leading to the acquisition of a stellate morphology, which resembles that of astrocyte in vivo [26,27,28]. The addition of LPA has been shown to reverse astrocyte stellation and extensive evidence indicates that the reversal is mediated by the activation of RhoA [29,30,31]. We therefore examined the effects of amitriptyline on astrocyte stellation induced by the β-adrenergic agonist l-isoproterenol. To differentiate a stellate morphology from the polygonal shape usually adopted by cultured astrocytes we employed staining for glial fibrillary acidic protein (GFAP) [29]. As shown in Figure 5, the addition of LPA (1 µM) to l-isoproterenol-treated astrocytes decreased the percentage of stellate cells by 95 ± 4% (*p* < 0.001, Student’s *t* test), whereas the addition of amitriptyline (10 µM) produced no significant reversal of astrocyte stellation. When amitriptyline was combined with LPA, it reduced the effect of LPA by 29 ± 14% (*p* < 0.05, Student’s *t* test).

### 2.6. Amitriptyline Attenuates Stress Fiber Formation and Focal Adhesion Kinase (FAK) Phosphorylation Induced by LPA

**Figure 6:**
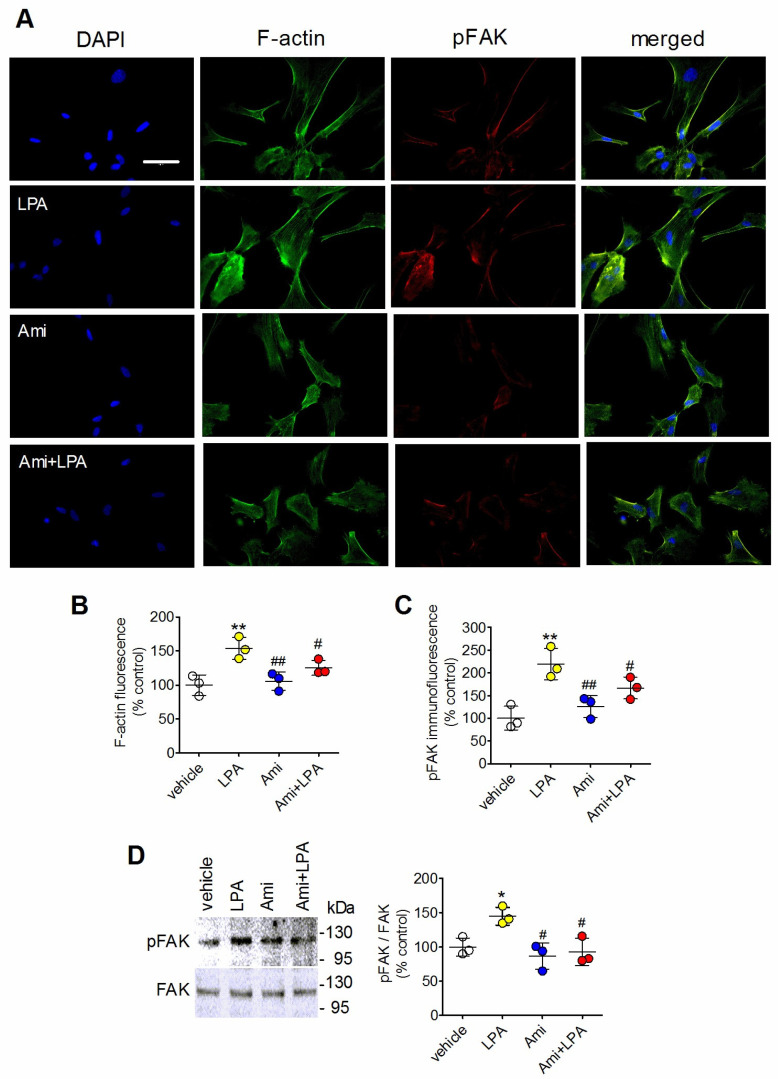
Amitriptyline counteracts LPA-induced stress fiber accumulation and FAK phosphorylation (pFAK) in astrocytes. (**A**) Serum-starved astrocytes were incubated for 5 min with either vehicle or 10 µM amitriptyline (Ami) and then treated for 90 min with either vehicle or 1 µM LPA. Cells were analyzed for F-actin (green color) and pFAK (red color) staining by fluorescence microscopy. Nuclei were stained with DAPI (blue color). Bar = 50 µm. (**B**,**C**) The values of integrated densities of F-actin (**B**) and pFAK (**C**) are reported as percent of control and are the mean ± SD of three independent experiments. (**D**) Serum-starved astrocytes were incubated for 5 min with either vehicle or 10 µM amitriptyline and then treated for 10 min with either vehicle or 1 µM LPA. Cell lysates were analyzed for pFAK and FAK levels by Western blot. The position of molecular weight standards is reported on the right side of the immunoblots. Values are the mean ± SD of three independent experiments. * *p* < 0.05, ** *p* < 0.01 vs. control (vehicle-treated cells); ^#^ *p* < 0.05, ^##^ *p* < 0.01 vs. LPA alone by Anova followed by Newman–Keuls test.

In different cell types, including astrocytes, Rho signaling-induced morphological changes involve induction of cellular contractility, assembly of stress fibers and focal adhesions [29,32,33,34,35]. We investigated the effects of amitriptyline on stress fiber formation in comparison with LPA (Figure 6A). In line with previous studies [29,36], exposure of astrocytes to LPA (1 µM) resulted in an accumulation of stress fibers, as indicated by the increase in F-actin staining (54 ± 16%, *p* < 0.01) (Figure 6A,B). Conversely, amitriptyline (10 µM) was without effect and, when associated with LPA, reduced the response to the lysophospholipid by 56 ± 15% (*p* < 0.05, Student’s *t* test) (Figure 6A,B).

The activation of FAK through autophosphorylation at Tyr397 has been shown to promote the formation of stress fibers and focal adhesions in cooperation with Rho signaling [36,37]. Immunofluorescence analysis (Figure 6A,B) showed that treatment of rat astrocytes with LPA (1 µM) increased phospho-FAK immunoreactivity by 119 ± 34% (*p* < 0.01), as compared to vehicle-treated cells, whereas no significant changes were induced by amitriptyline. Phospho-FAK staining in astrocytes treated with the combination of amitriptyline with LPA was 52 ± 6% lower (*p* < 0.05, Student’s *t* test) than that detected in cells treated with LPA alone (Figure 6C).

Western blot analysis of phospho-FAK detected an immunoreactive band of approximately 120 kDa, which corresponded to the molecular mass of FAK (Figure 6D). Exposure to LPA (1 µM) increased phospho-FAK levels by 46 ± 13% (*p* < 0.05), whereas amitriptyline (10 µM) had no significant effect *per se*, but completely inhibited the stimulation by LPA.

### 2.7. Amitriptyline Does Not Affect MYPT1 Phosphorylation and Inhibits the Stimulation Induced by LPA

**Figure 7:**
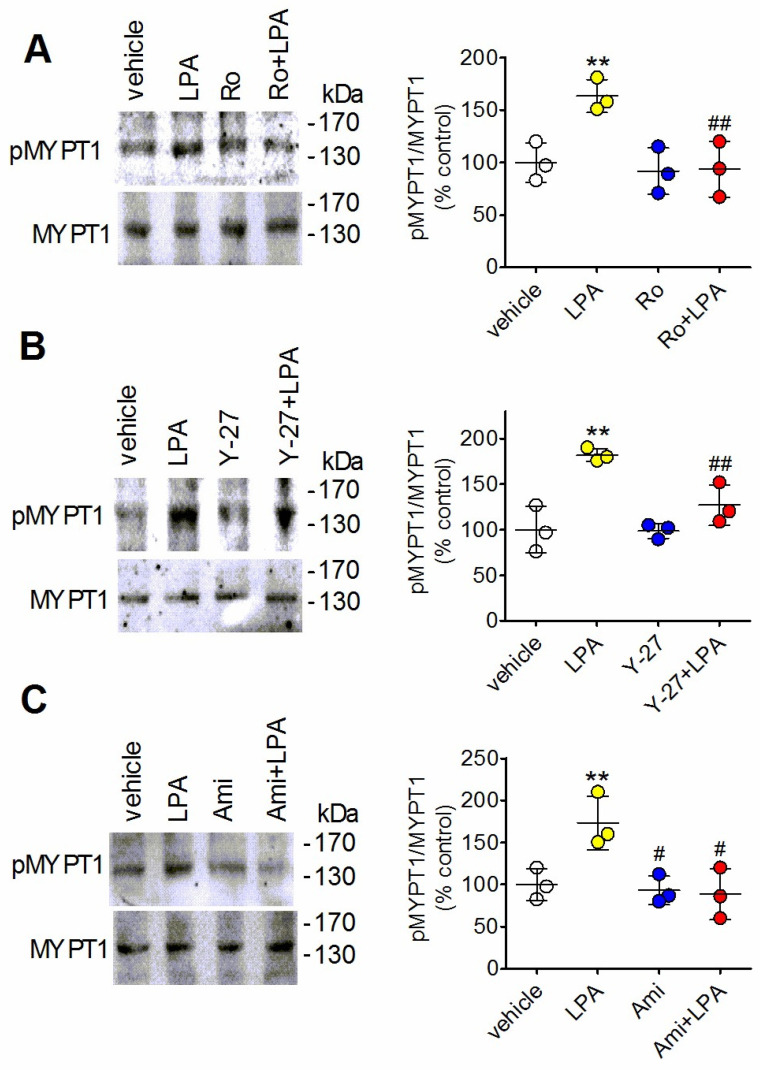
Amitriptyline inhibits LPA-induced MYPT1 phosphorylation in astrocytes. Serum-starved cells were incubated for 20 min with either vehicle or 100 nM Ro-6842262 (Ro) (**A**), for 60 min with either vehicle or 15 µM Y-27632 (Y-27) (**B**), and for 5 min with either vehicle or 10 µM amitriptyline (Ami) (**C**). Cells were then treated for 10 min with either vehicle or 1 µM LPA. Cell lysates were analyzed for phospho-MYPT1 and MYPT1 immunoreactivity by Western blots. The position of molecular weight standards is indicated on the right side of the immunoblots. Values are the mean ± SD of three independent experiments. ** *p* < 0.01 vs. control (vehicle-treated cells); ^#^ *p* < 0.05. ^##^ *p* < 0.01 vs. LPA alone by Anova followed by Newman–Keuls test.

The myosin target subunit of myosin phosphatase isoform 1 (MYPT1) is a widely expressed component of the myosin phosphatase holoenzyme, which regulates the interaction between myosin and actin [38]. Activated Rho triggers myosin phosphatase inhibition and cellular contractility through the Rho-associated kinase (ROCK)-induced phosphorylation of MYPT1 at Thr696 and Thr853 [39]. To further investigate the differential actions of LPA and amitriptyline on Rho signaling we examined the effects of these agents and their combination on the levels of phospho-MYPT1 in astrocytes. As shown in Figure 7A a brief exposure to LPA (1 µM) caused a significant increase in MYPT1 phosphorylation (73 ± 19%, *p* < 0.01), which was completely blocked by the LPA_1_ receptor antagonist Ro-6842262 (100 nM). The stimulatory effect of LPA was prevented by preincubating the astrocytes with the ROCK inhibitor Y-27632, implying the involvement of ROCK activity (Figure 7B). When the effect of amitriptyline was examined, it was found that the antidepressant failed to affect phospho-MYPT1 levels and when combined with LPA completely inhibited the stimulatory effect of the phospholipid (Figure 7C).

## 3. Discussion

The finding that different classes of antidepressants can directly act at LPA receptors raised the important question as to whether these drugs could affect the cellular responses triggered by LPA. Astroglial cells, which express different LPA receptor subtypes [8,40], are known to be a relevant central target of antidepressants [41,42,43], and altered astroglial functions have been associated with depressive-like behavior [44,45]. Therefore, astroglial cells represent a congruent cellular system where we can investigate the functional interaction between LPA and antidepressants.

The present study shows that in astroglial cells amitriptyline, a widely used antidepressant, has the ability to differentially modulate LPA receptor-mediated responses by enhancing the stimulation of ERK1/2 signaling on the one hand, and curtailing Rho activation and the associated morphological and molecular events on the other.

Previous studies have shown that in astroglial cells LPA and antidepressants can activate ERK1/2 predominantly through LPA_1_ receptors coupled to pertussis toxin-sensitive G_i/o_ proteins [8,46]. Here, we show that amitriptyline potentiated the LPA-induced phosphorylation/activation of distinct components of the ERK1/2 signaling pathway both upstream and downstream of ERK1/2. Thus, the facilitatory input exerted by amitriptyline appeared to propagate from the plasma membrane, where it potentiated FGF receptor phosphorylation, to the cell nucleus, where it was translated into a greater CREB activation. An outcome of the synergistic interaction was found to be the enhancement of BDNF expression, likely as a consequence of the increased CREB activation. CREB-BDNF signaling is well known to be implicated in the pathophysiology of depression and in the generation of antidepressant-like responses [47,48,49]. Astroglial BDNF is considered to play a crucial role in modulating astrocyte–neuron communication [50]. Thus, there is evidence that astrocyte-derived BDNF can promote synapse formation [51] and regulate long-term synaptic plasticity and memory consolidation at excitatory synapses [52]. BDNF has also been shown to stimulate GAT1-mediated GABA uptake in astrocytes, thereby increasing the synaptic clearance of GABA [53]. Considering this information, the present findings suggest that by potentiating LPA-induced ERK1/2-CREB signaling in astroglia amitriptyline may enhance the synaptic availability of BDNF and modulate neurotransmission at neuronal circuits controlling mood and emotion.

LPA has been shown to trigger the activation of the small GTPase Rho by promoting the receptor coupling to the G proteins G_12/13_ [3,4,5,6]. Amitriptyline, at a concentration (10 µM) that produced a significant stimulation of G_i/o_-mediated ERK1/2 signaling, failed to induce Rho activation and impaired the stimulatory effect of LPA. The antidepressant was also found to have no effect on several cellular responses known to be dependent on Rho activation, including the reversal of astrocyte stellation, accumulation of stress fibers, and induction of FAK and MYPT1 phosphorylation. In addition, amitriptyline reduced the LPA stimulation of these responses, indicating that its inhibitory effect extended beyond the control of Rho activation to affect intracellular actin filament assembly, ROCK-dependent regulation of actomyosin interaction, and the dynamic of astrocyte morphology.

At present the pathophysiological implications of the amitriptyline negative control on LPA-activated Rho–ROCK signaling remain to be defined. It is generally recognized that astrocyte morphology plays a crucial role in modulating synaptic and neuronal function through reciprocal signaling [54]. The extent of synaptic coverage by astrocyte processes is a critical factor in determining the efficacy of signal exchange and may be altered by changes in astrocyte morphology involving retraction or loss of astrocyte processes [35]. It is possible that increased concentrations of LPA which occur during brain injury may alter the astrocyte–neuron interaction by enhancing Rho–ROCK signaling. It has been reported that in organotypic slice culture of the hippocampus, adenoviral expression of RhoA caused a loss of astrocyte processes [55]. Conversely, it has been shown that in *Drosophila* astrocytes, suppression of focal adhesion molecules under conditions of neuronal hyperactivity enhances astrocyte synaptic coverage and the expression of glutamate transporters [56]. It is noteworthy that several preclinical studies have highlighted the beneficial effects of inhibiting Rho–ROCK signaling in preventing stress-induced synaptic alterations and promoting antidepressant-like activity [57]. In light of these data, one may speculate that amitriptyline, by counteracting LPA-induced Rho/ROCK signaling, may stabilize the astrocyte–neuron interaction and preserve the astrocyte modulatory function on synaptic transmission.

A limitation of this study is that it is conducted in vitro and does not examine the interactions between different cell types which occur in vivo. Moreover, it is not known whether LPA-activated Rho signaling affects astroglial morphology in vivo as it does in vitro.

The present demonstration that amitriptyline exerts opposite effects on LPA signaling in astroglial cells provides further support to the idea that the antidepressant behaves as an allosteric ligand of LPA receptors. In fact, a key feature of GPCR allosteric ligands is that they can act as positive and negative modulators as a function of the signaling pathway recruited [58]. Amitriptyline acts as a positive allosteric modulator of G_i/o_-mediated ERK1/2 signaling by both displaying agonist activity and enhancing LPA stimulation. Conversely, the drug appears to be a negative allosteric modulator of the receptors when LPA triggers G_12/13_-mediated Rho–ROCK activation. Interestingly, a recent study of the cryo-electron microscopy structures of LPA-activated LPA_1_ receptor in complex with either G_i_, G_q_ or G_13_ found a differential requirement of the intracellular loop 2 of the receptor for the activation of G_i_ and G_13_ [59]. Thus, it is conceivable that the binding of amitriptyline to an allosteric site may promote a conformational state of LPA-activated receptors that favor coupling to one G protein over another. Notwithstanding that additional studies are necessary to identify the allosteric site targeted by amitriptyline [60], the present findings suggest that the differential regulation of astroglial LPA signaling with amplification of the ERK1/2-CREB-BDNF axis to promote neuroprotection and neural plasticity, and inhibition of Rho–ROCK-mediated morphological alterations leading to impaired astrocyte–neuron communication, may constitute an important novel component of the mechanism of action of amitriptyline and possibly of other antidepressant drugs.

## 4. Materials and Methods

### 4.1. Materials

Amitriptyline hydrochloride, l-isoproterenol hydrochloride and 4′-6-diamidino-2-phenylindole dihydrochloride (DAPI) were purchased from Sigma Aldrich (St. Louis, MO, USA). 1-Oleoyl-lysophosphatidic acid (LPA) and Y-27632 dihydrochloride were obtained from Santa Cruz Biotechnology (Dallas, TX, USA). Ro-6842262 and PD98059 were from Tocris Bioscience (Bristol, UK). AM966 was purchased from Chem Scene (Monmouth Junction, NJ, USA).

### 4.2. Cell Culture

C6 rat glioma cells (European Collection of Cell Cultures, Porton Down, Wiltshire, UK) were grown in Ham’s F12 medium (EuroClone, Pero, Italy) containing 2 mM L-glutamine, 10% fetal calf serum (FCS), and 1.0% penicillin/streptomycin (P/S) (Sigma Aldrich) at 37 °C in a humidified atmosphere of 5% CO_2_ in air. The medium was renewed every other day. When they reached sub-confluency, the cultures were passaged by a brief wash with Dulbecco’s phosphate-buffered saline (EuroClone) followed by incubation for 5 min with 0.05% Trypsin–EDTA solution (Sigma Aldrich).

Frozen aliquots of astrocytes, prepared from cerebral cortex of newborn rats [8] and stored in liquid nitrogen, were thawed and used to prepare primary cultures. Astrocytes were grown in Dulbecco’s modified Eagle’s (EuroClone)–Ham’s F12 medium (1:1) supplemented with 10% FCS and 1% P/S in an atmosphere with 5% CO_2_. Sub-confluent cell cultures were detached using cell dissociation solution (Sigma Aldrich) followed by 0.25% Trypsin–EDTA (Sigma Aldrich).

### 4.3. Cell Treatment

Unless otherwise specified, cells were seeded in six-well plates and used when they reached sub-confluency. Cells were serum-starved for 24 h, the medium was renewed, and the cells were incubated for 1 h before treatment with the test agents as indicated in the text. Stock solution of the drugs was prepared as follows: amitriptyline, l-isoproterenol and Y-27632 10 mM in H_2_O; LPA 23 mM in ethanol; Ro-6842262, AM966 and PD98059 10 mM in dimethyl sulfoxide (DMSO). Dilutions of LPA, Ro-6842262, AM966 and PD98059 were made in DMSO. The final concentration of DMSO was 0.5%. Control samples were treated with equal amounts of vehicles. Drug concentrations and incubation times were chosen on the basis of the results obtained in previous studies [8,13,21].

The amitriptyline concentrations used (1 and 10 µM) are higher than the therapeutic plasma concentrations (100–250 ng/mL) but are consistent with the brain concentrations reached by the drug, which shows a brain-to-blood ratio as high as 20–40:1 [61,62].

### 4.4. Rho Activation Assay

Rho activation was examined by using a commercial kit obtained from Cell Signaling Technology, Beverly, MA, USA (cat. no. 8820). Following exposure to the test agents, cells were washed with ice-cold PBS and lysed by the addition of a lysis buffer containing 1 mM phenylmethylsulphonyl fluoride (PMSF). Cells were scraped, vortexed, and incubated at ice-bath temperature for 5 min. Samples were then centrifuged at 16,000× *g* for 15 min at 4 °C and the supernatants were assayed for protein concentration. An aliquot of each sample containing an equal amount of protein (approximately 500 µg) was immediately mixed with glutathione S-transferase-tagged rhotekin Rho-binding domain coupled to glutathione resin in a spin cup and incubated for 1 h at 4 °C. Unbound proteins were removed by centrifugation at 6000× *g* for 30 s. Following repetitive washing with lysis buffer, samples containing activated Rho were eluted by the addition of 2x SDS sample buffer supplemented with 200 mM dithiothreitol and heated at 100 °C for 5 min. An aliquot of the supernatant of each sample was taken prior to precipitation for determination of total Rho. Samples were analyzed by Western blot.

### 4.5. Western Blot Analysis

Following treatment, cells were washed in ice-cold phosphate-buffered saline (PBS) (pH 7.4) and scraped in ice-cold RIPA buffer containing PBS, 4 mM sodium pyrophosphate, 2 mM sodium orthovanadate, 10 mM sodium fluoride, 20 nM okadaic acid, 0.5% phosphatase inhibitor cocktail 3, 1% protease inhibitor cocktail (Sigma Aldrich), 1 mM PMSF, 0.1% sodium dodecyl sulphate (SDS), 1% Nonidet P-40 and 0.5% sodium deoxycholate (pH 7.4). Cell lysates were subjected to sonication for 5 s at ice-bath temperature, and the protein concentration was determined by the Bio-Rad Protein assay kit (Bio-Rad Lab., Hercules, CA, USA). Aliquots of the cell lysates containing an equal amount of protein (10–15 µg/lane) were mixed with 5x Laemmli sample buffer and loaded onto either home-made 10 and 12.5% polyacrylamide gels or pre-casted Nu-PAGE Bis-Tris gels (10 and 4–12%, Invitrogen/Thermo Fisher Scientific, Monza, Italy). Following separation by electrophoresis, cell proteins were electrophoretically transferred to either polyvinylidene difluoride membranes (Immobilon-P, Merck Millipore, Darmstadt, Germany) or nitrocellulose membranes (Amersham Biosciences, Piscataway, NJ, USA) using a Semiphor Transphor semi-dry unit (GE Healthcare, Little Chalfont, Buckinghamshire, UK). Membranes were blocked with 5% non-fat dry milk (Santa Cruz Biotechnology), washed and incubated overnight at 4 °C with one of the following primary antibodies: rabbit monoclonal anti-phospho-MEK1/2 (Ser217/221) (cat. no. 9154, Cell Signaling Technology, Beverly, MA, USA) (1:1000), mouse monoclonal anti-MEK1/2 (sc-81504, Santa Cruz Biotechnology) (1:1000), rabbit polyclonal anti-phospho-ERK 1(Thr202/Tyr204)/ERK2 (Thr185/Tyr187) (cat. no. RA15002, Neuromics, Northfield, MN, USA) (1:1000), rabbit polyclonal anti-ERK1/2 (cat. no. 9102, Cell Signaling Technology) (1:1000), mouse monoclonal anti-phospho-FGF receptor (Tyr653/654) (cat. no. 3476, Cell Signaling Technology) (1:1000), rabbit polyclonal anti-FGF receptor 1 (cat. no. 3472, Cell Signaling Technology) (1:1000), rabbit polyclonal anti-phospho-Elk-1 (Ser383) (cat. no. 9181, Cell Signaling Technology) (1:1000), mouse monoclonal anti-Elk-1 (Sc-365876, Santa Cruz Biotechnology) (1:1000), rabbit monoclonal anti-phospho-CREB (Ser133) (cat. no. 9198, Cell Signaling Technology) (1:1000), rabbit monoclonal anti-CREB (cat. no. 9197, Cell Signaling Technology) (1:1000), chicken IgY anti-BDNF (cat. no. G1641, Promega, Madison, WI, USA) (1:500), rabbit polyclonal anti-BDNF (sc-546, Santa Cruz Biotechnology) (1:500), rabbit polyclonal anti-phospho-MYPT1 (Thr696) (cat. no. 5163, Cell Signaling Technology) (1:1000), rabbit polyclonal anti-MYPT1 (cat. no. 2634, Cell Signaling Technology) (1:1000), mouse monoclonal anti-phospho-FAK (Tyr397) (sc-81493, Santa Cruz Biotechnology) (1:1000), rabbit polyclonal anti-FAK (cat. no. 3285, Cell Signaling Technology) (1:1000), rabbit polyclonal anti-Rho (cat. no. 8789, Cell Signaling Technology) (1:667), rabbit polyclonal anti-GAPDH (cat. no. 247002g, Synaptic Systems, Gottingen, Germany) (1:2000). Membranes were then washed and incubated with either horseradish peroxidase (HRP)-conjugated AffiniPure goat anti-rabbit IgG (cat. no. 111-035-003, Jackson ImmunoResearch, Westgrove, PA, USA) (1:2000), purified recombinant mouse IgGk light chain binding protein conjugated to HRP (sc-516102, Santa Cruz Biotechnology) (1:1000), or HRP-conjugated goat anti-IgY Fc antibody (cat. no. GAYFC-HRP, GeneWay, San Diego, CA, USA) (1:5000) for 1 h at room temperature. Immunoreactive bands were detected by using Clarity Western ECL substrate (Bio-Rad Lab.) and digital images were acquired by using IBright 1500 gel imager (Invitrogen/Thermo Fisher Scientific). The size of the immunoreactive bands was determined by using molecular weight standards (prestained protein SharpMass VII; Euroclone, Milan, Italy). Band densities were measured by using the NIH ImageJ software (1.54r) (US National Institutes of Health, Bethesda, MD, USA). The density of the phosphorylated protein bands was normalized to the density of the corresponding total protein in the same sample, whereas active Rho and BDNF were normalized to the density of total Rho and GAPDH, respectively.

### 4.6. Immunofluorescence Microscopy

Cells were plated and grown on glass coverslips (Electron Microscopy Sciences, Hatfield, PA, USA) precoated with 0.01% poly-D-lysine (Gibco/Thermo Fisher Scientific) to 50–60% confluency in 24-well plates. Following incubation in serum-free medium for 24 h, the medium was renewed and the cells were treated with the test agents as specified in the text. Thereafter, cells were washed, fixed with ice-cold 4% formaldehyde for 45 min and permeabilized with 0.2% Triton X-100 for 5 min. Following blockade with 3% bovine serum albumin and 1% normal goat serum for 1 h, cells were incubated overnight at 4 °C with the following primary antibodies: rabbit polyclonal anti-phospho-CREB (Ser133) (cat. no. 06-519, Upstate Biotechnology Inc., Lake Placid, NY, USA) (1:400), rabbit polyclonal anti-BDNF (sc-546, Santa Cruz Biotechnology) (1:100), mouse monoclonal anti-α-tubulin (sc.5286, Santa Cruz Biotechnology) (1:100), rabbit polyclonal anti-GFAP (cat. no. G9269, Sigma Aldrich) (1:200) and mouse monoclonal anti-phospho-FAK (Tyr397) (sc-81493, Santa Cruz Biotechnology) (1:100). After washing, cells were incubated with either Alexa-Fluor488-conjugated goat anti-rabbit IgG (H+L) (cat. no. A11034, Molecular Probes/Life Technologies, Eugene, OR, USA) (1:1500) or Alexa-Fluor Plus 555-conjugated goat anti-mouse IgG (H+L) (Invitrogen/Thermo Fisher Scientific) (1:1000). To stain the polymerized form of actin (F-actin) cells were incubated with 100 nM actin-stained 488 fluorescent phalloidin (cat. no. PHDG1, Cytoskeleton Inc., Denver, CO, USA). Cell nuclei were stained with 0.1 µg/mL DAPI. Fluorescence staining was visualized by using an EVOS M5000 imaging system (Invitrogen/Thermo Fisher Scientific) equipped with light cubes for DAPI (AMEP4950), GFP (AMEP4951) and RFP (AMEP4952), and 20–40x fluorite objectives. Digital images were acquired using constant camera settings within each experiment and were analyzed using the NIH ImageJ software.

For quantification of phospho-CREB fluorescence, the integrated density of the green staining was measured within the region of the cell nucleus and in an adjacent area, which was used as background value. Cells were deemed to be positive if the integrated density corrected for the background was equal or above a threshold value corresponding to one standard deviation above the integrated density of the respective control (vehicle-treated) samples.

For quantification of BDNF, F-actin and phospho-FAK stainings, images obtained in each fluorescence channel were thresholded and the integrated densities of BDNF, F-actin and phospho-FAK stainings were normalized to that of DAPI in the same image. No labeling was detected in a parallel set of samples treated with preimmune IgG. Images were analyzed by an investigator unaware of the treatment.

### 4.7. Statistical Analysis

Results are reported as means ± SD of the indicated number of independent experiments. Statistical analysis was performed by using the program Graph Pad Prism 5 (San Diego, CA, USA), which was also used to calculate EC_50_ and E_max_ values. Unless otherwise indicated, data are expressed as percentage or fold stimulation of control, which was included in each independent experiment. The control group was set as 100 or 1 with a variance obtained by expressing each control value as a percentage of the mean of the raw values of the control group. The outcome (O) of the combination of amitriptyline with LPA was calculated as follows: O = Δ Ami + LPA − Δ Ami/Δ LPA, where Δ Ami + LPA, Δ Ami and Δ LPA are the net stimulations (calculated as percent of control) induced by the combination of amitriptyline with LPA, and by amitriptyline and LPA alone, respectively. O = 1 indicates additivity, O > 1 indicates positive interaction, O < 1 indicates negative interaction. Statistical significance between experimental groups was assessed by either analysis of variance (Anova) followed by Newman–Keuls multiple comparison test or unpaired Student’s *t* test, as appropriate. A value of *p* < 0.05 was considered as the level of statistical significance.

## Data Availability

The data presented in this study are included in the article/Supplementary Materials. Further inquiries can be directed to the corresponding author.

## Supplementary Materials

The following supporting information can be downloaded at: https://www.mdpi.com/article/10.3390/ijms27083660/s1.

## Abbreviations

The following abbreviations are used in this manuscript:

AM	AM966
Ami	amitriptyline
BDNF	brain-derived neurotrophic factor
CREB	cyclic AMP response element binding protein
DAPI	4′-6-diamidino-2-phenylindole
DMSO	dimethyl sulphoxide
ERK1/2	extracellular signal-regulated protein kinases 1 and 2
FAK	focal adhesion kinase
FGFR	fibroblast growth factor receptor
GAPDH	glyceraldehyde-3 phosphate dehydrogenase
GFAP	glial fibrillary acidic protein
GPCRs	G protein-coupled receptors
LPA	lysophosphatidic acid
MYPT1	myosin target subunit of myosin phosphatase isoform 1
PD	PD98059
Ro	Ro-6842262
ROCK	Rho-associated kinase
Y-27	Y-27632
