# Supplementary material for: The Antidepressant Amitriptyline Upregulates ERK1/2 Signaling and Inhibits Rho-Mediated Responses Induced by Lysophosphatidic Acid in Astroglial Cells
Source: Int J Mol Sci. 2026 Apr 20;27(8):3660. doi: 10.3390/ijms27083660 (PMC13115648; doi:10.3390/ijms27083660)

Journal: *International Journal of Molecular Sciences*

Title: The antidepressant amitriptyline upregulates ERK1/2 signaling and inhibits Rho-mediated responses induced by lysophosphatidic acid in astroglial cells

Authors: M.C. Olianas, S. Dedoni and P. Onali

Supplementary information

Supplementary Figure 1

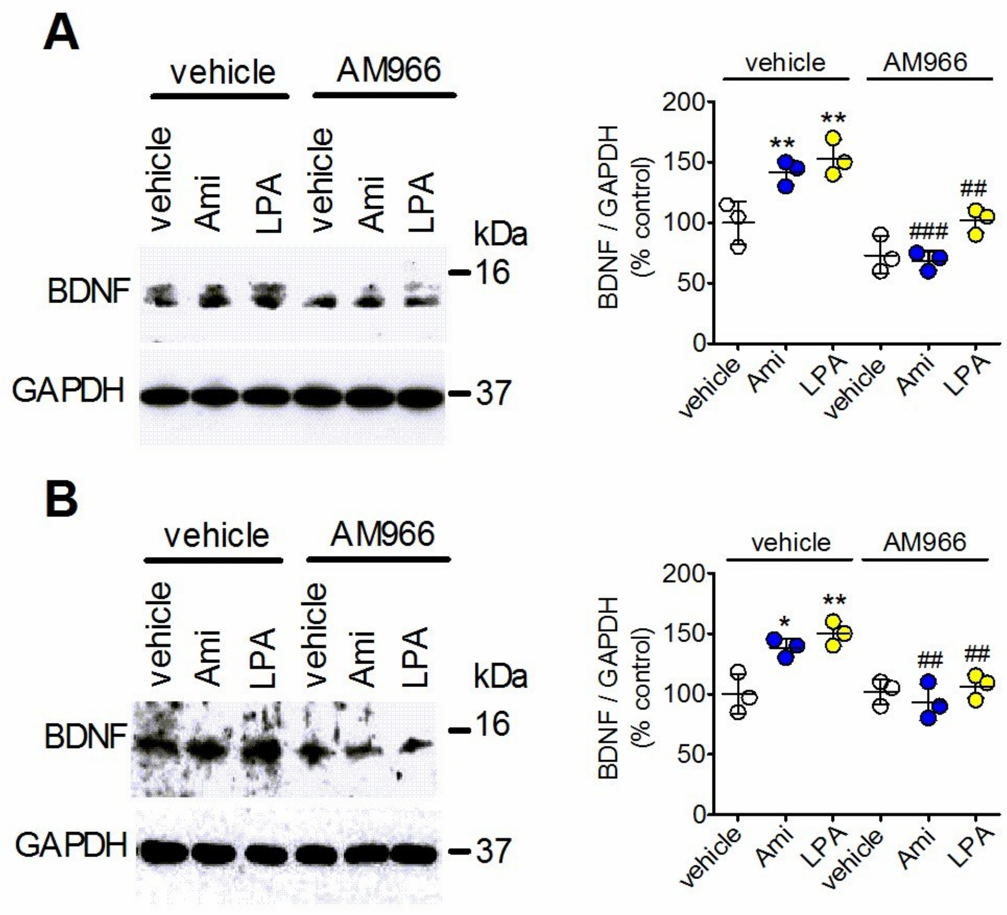

Figure S1. Induction of BDNF expression in astroglial cells by amitriptyline and LPA through LPA_1 receptors

Serum-starved C6 glioma cells (A) and rat astroglial cells (B) were preincubated for 10 min with either vehicle or 100 nM AM966 and then incubated for 24 h with either vehicle, 10 μM amitriptyline (Ami) or 10 μM LPA. Cell lysates were then analyzed for BDNF levels by Western blot using a primary antibody obtained from Promega. Values are the mean ± SD of three independent experiments. * $p < 0.05$, ** $p < 0.01$ vs control (vehicle-treated cells); ## p <0.01, ### $p < 0.001$ vs the corresponding treatment in the absence of antagonist by Anova followed by Neuman-Keuls test.

Journal: *International Journal of Molecular Sciences*

Title: The antidepressant amitriptyline upregulates ERK1/2 signaling and inhibits Rho-mediated responses induced by lysophosphatidic acid in astroglial cells.

Authors: M.C. Olianas, S. Dedoni and P. Onali

Supplementary information

Uncropped Western blots and original immunofluorescence images

Figure 1A

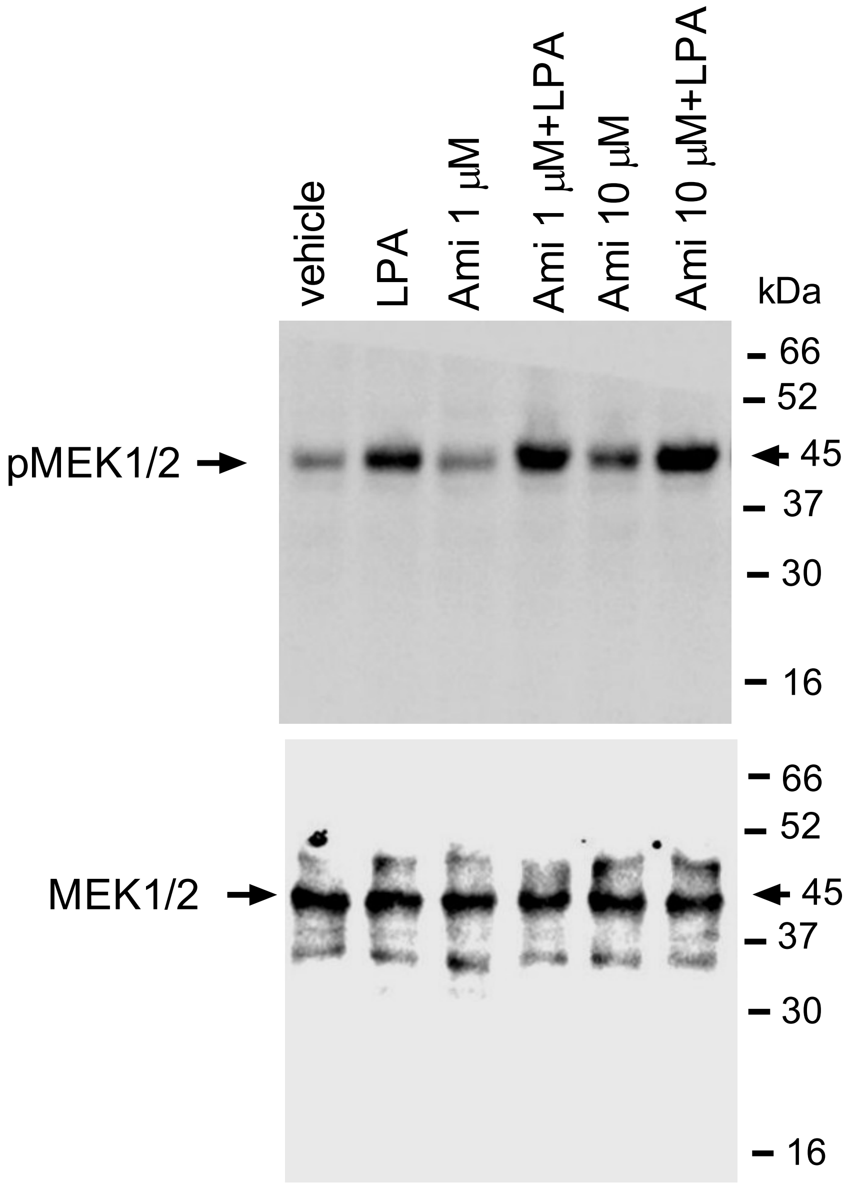

Figure 1B

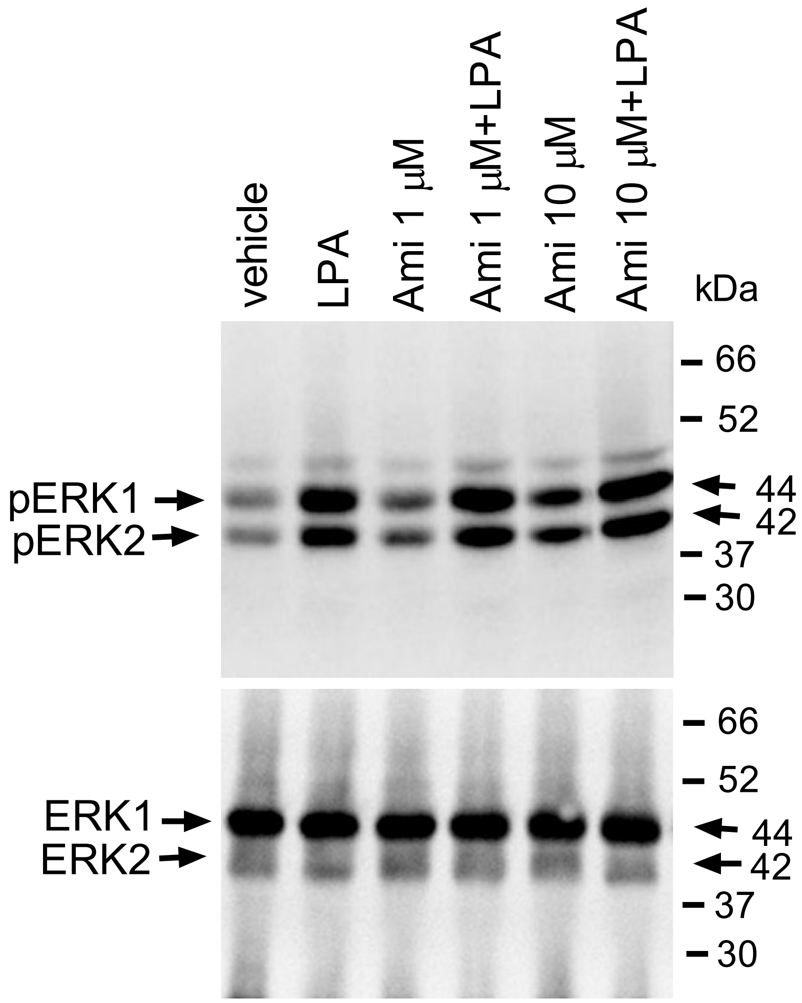
vehicle
LPA
Ami 1 μM
Ami 1 μM+LPA
Ami 10 μM
Ami 10 μM+LPA
kDa
66
52
pERK1
pERK2
44
42
37
30
66
52
ERK1
ERK2
44
42
37
30

Figure 1C

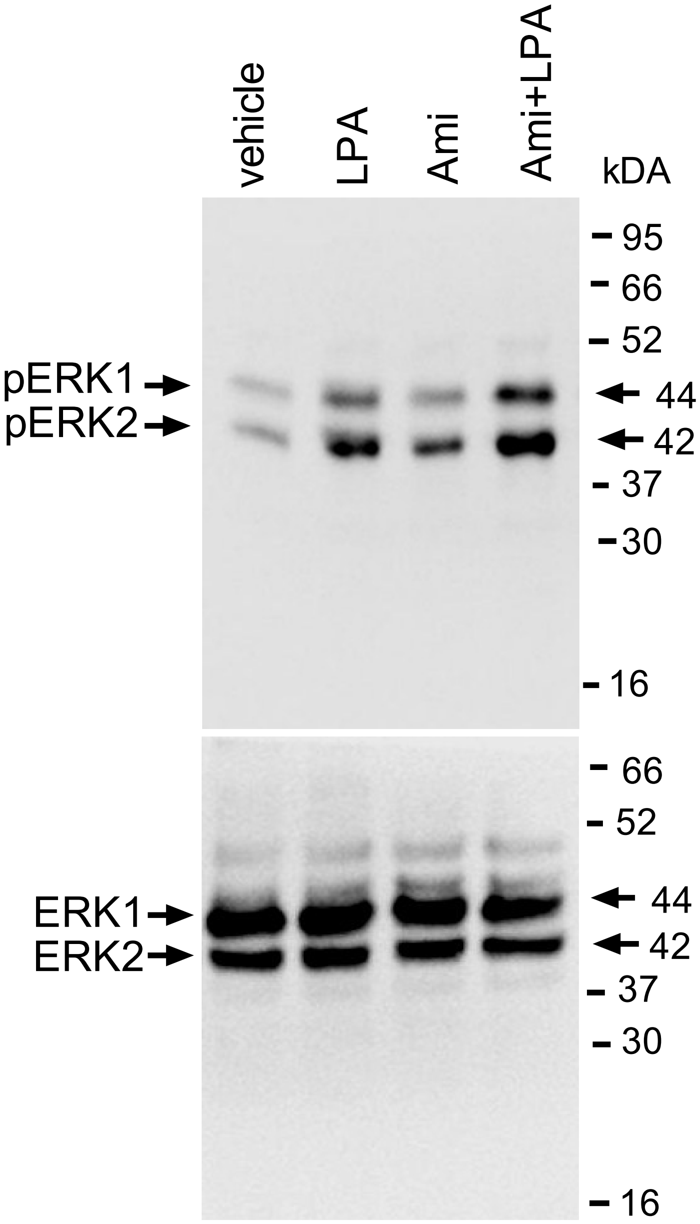
vehicle
LPA
Ami
Ami+LPA
kDA
95
66
52
pERK1
pERK2
44
42
37
30
16
66
52
ERK1
ERK2
44
42
37
30
16

Figure 1D

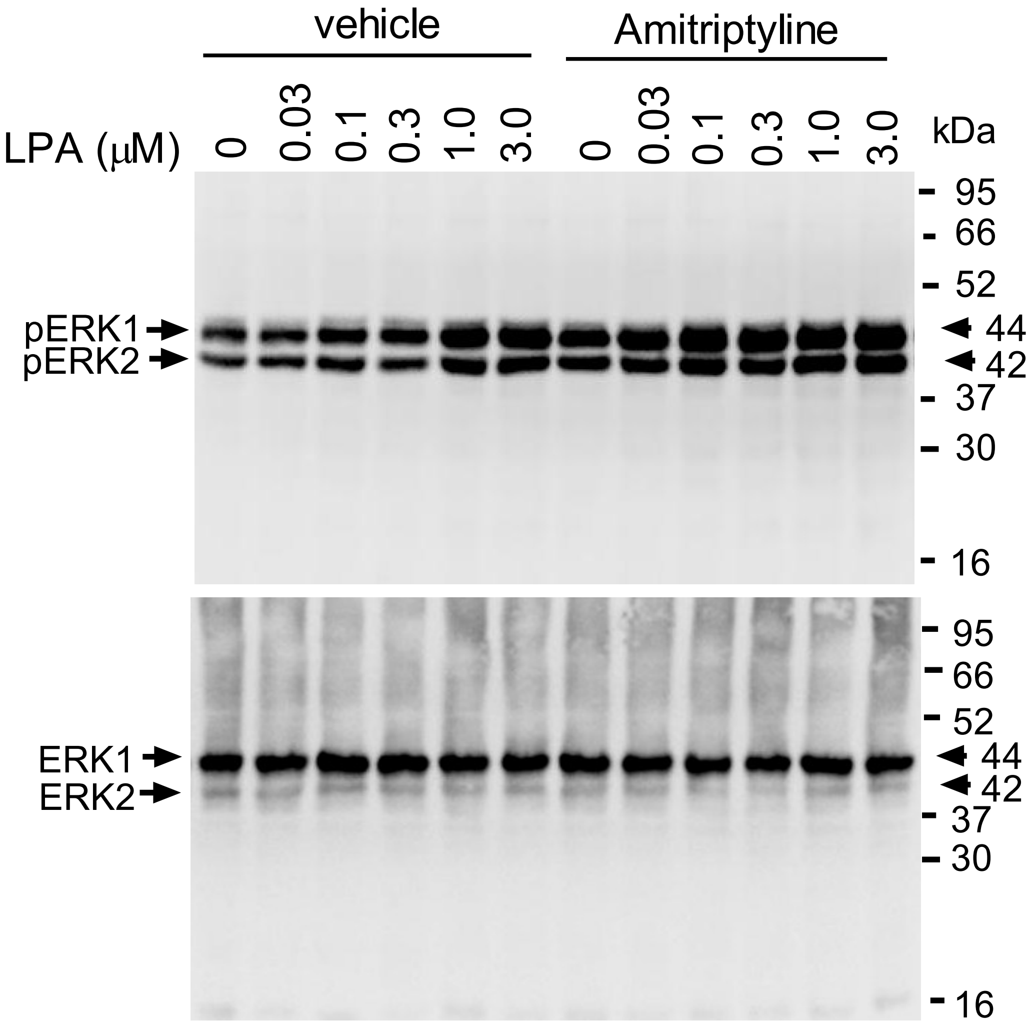
vehicle
Amitriptyline
LPA (μM)
0
0.03
0.1
0.3
1.0
3.0
0
0.03
0.1
0.3
1.0
3.0
kDa
95
66
52
pERK1
pERK2
44
42
37
30
16
95
66
52
ERK1
ERK2
44
42
37
30
16

Figure 1E

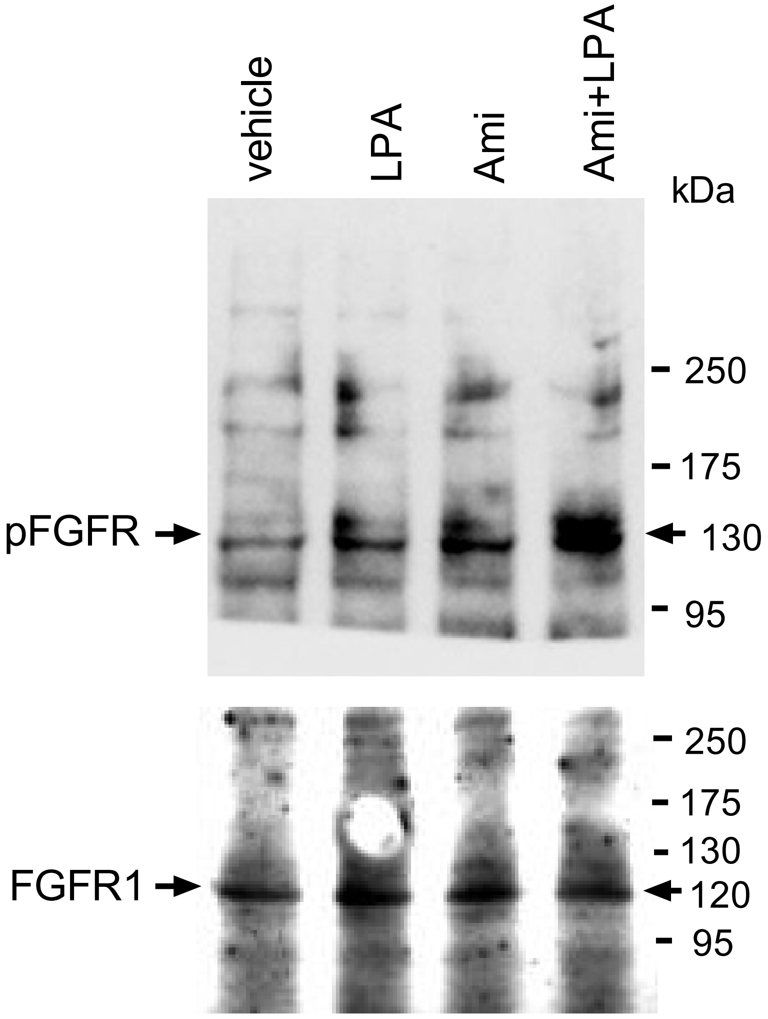
vehicle
LPA
Ami
Ami+LPA
kDa
250
175
130
95
pFGFR
250
175
130
120
95
FGFR1

Figure 1F

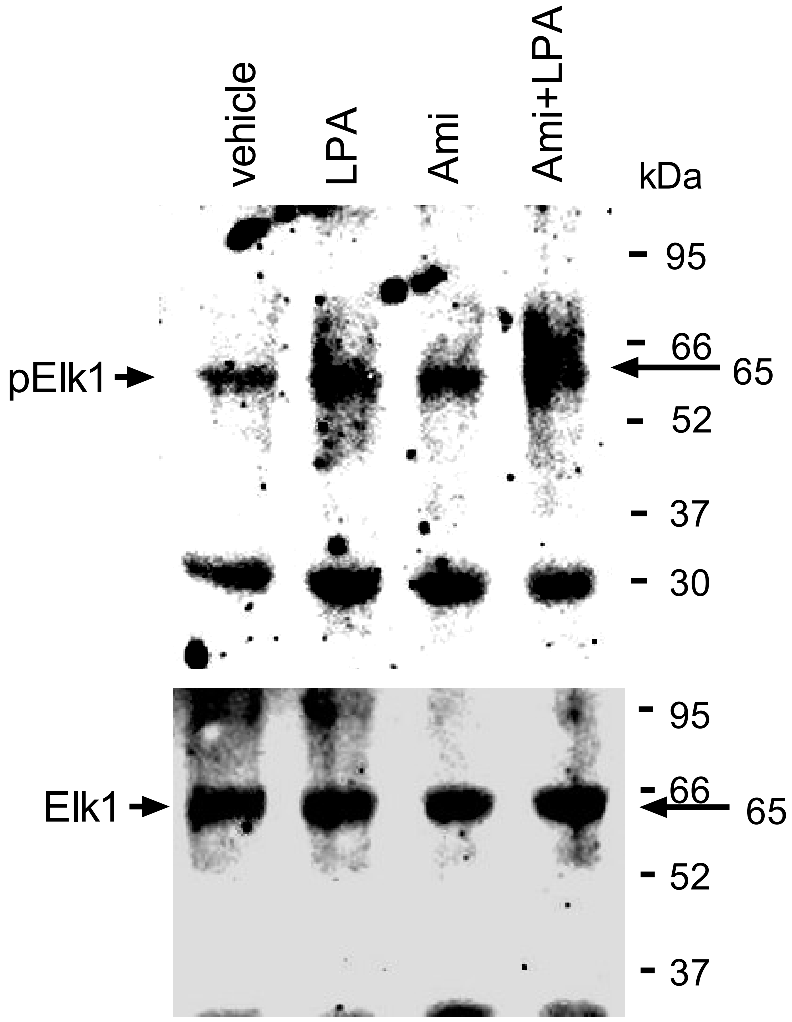

vehicle
LPA
Ami
Ami+LPA
kDa
95
66
65
52
37
30
pElk1
95
66
65
52
37
Elk1

Figure 2A

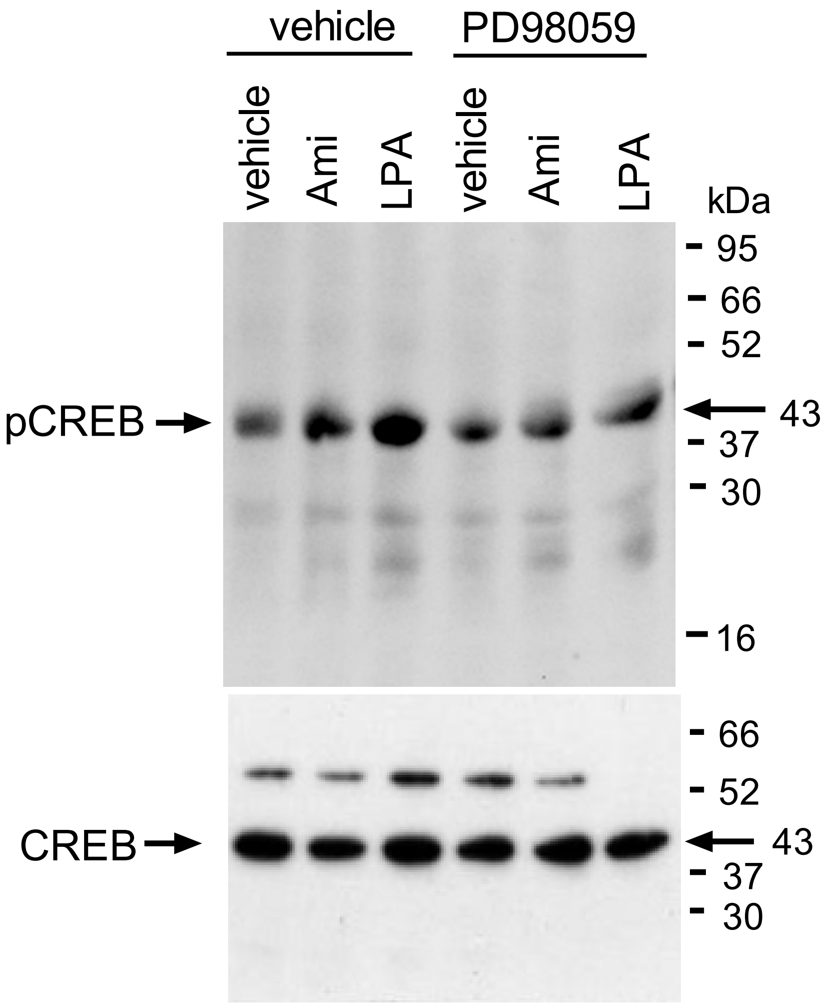
vehicle
PD98059
vehicle
Ami
LPA
vehicle
Ami
LPA
kDa
95
66
52
pCREB
43
37
30
16
66
52
CREB
43
37
30

Figure 2B

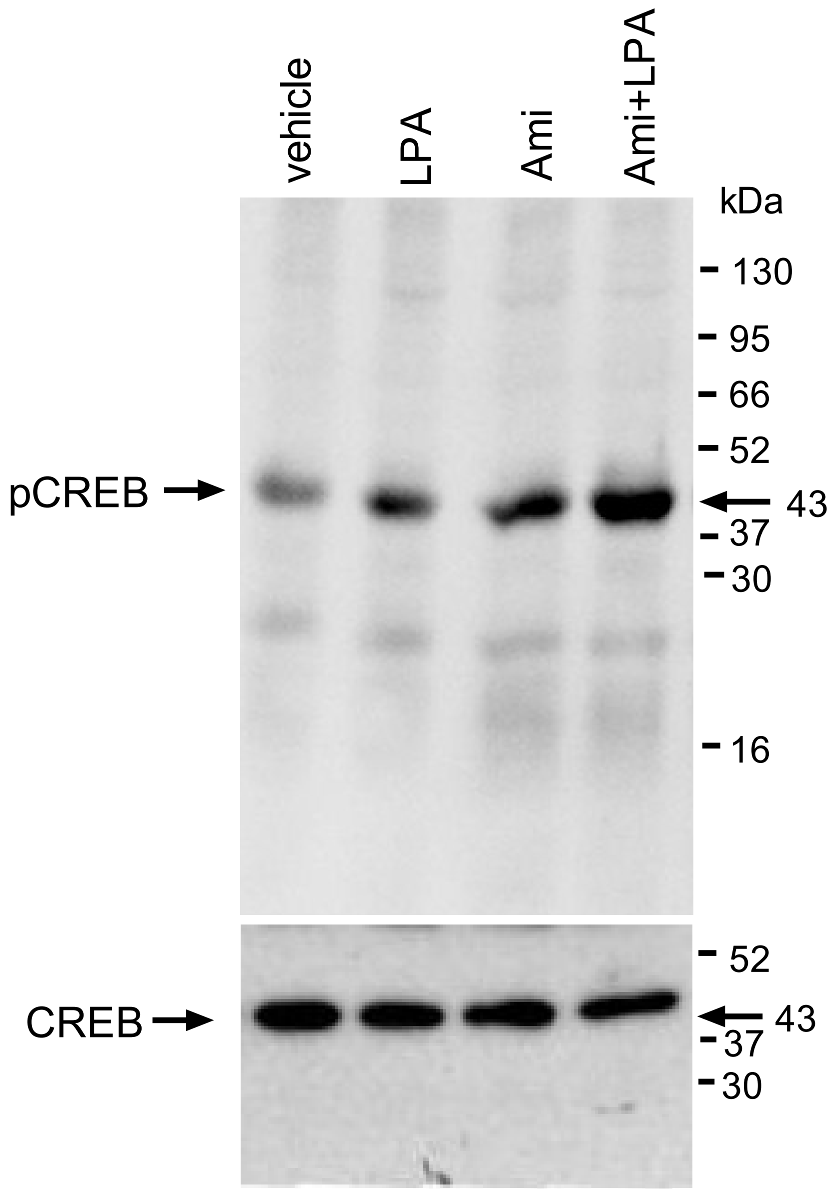

vehicle
LPA
Ami
Ami+LPA
kDa
130
95
66
52
43
37
30
16
pCREB
52
43
37
30
CREB

Figure 2C

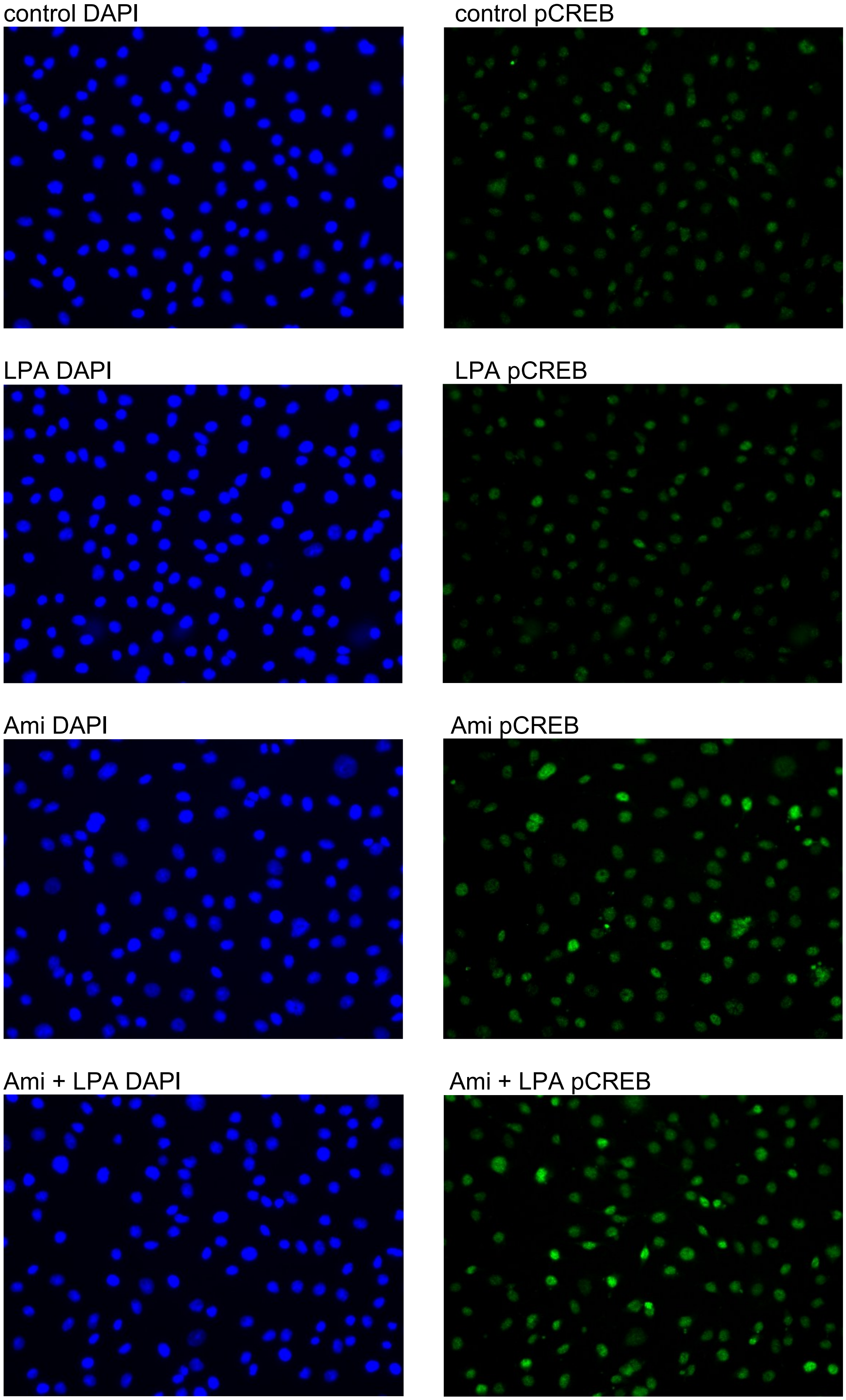
control DAPI
control pCREB
LPA DAPI
LPA pCREB
Ami DAPI
Ami pCREB
Ami + LPA DAPI
Ami + LPA pCREB
Figure 3A

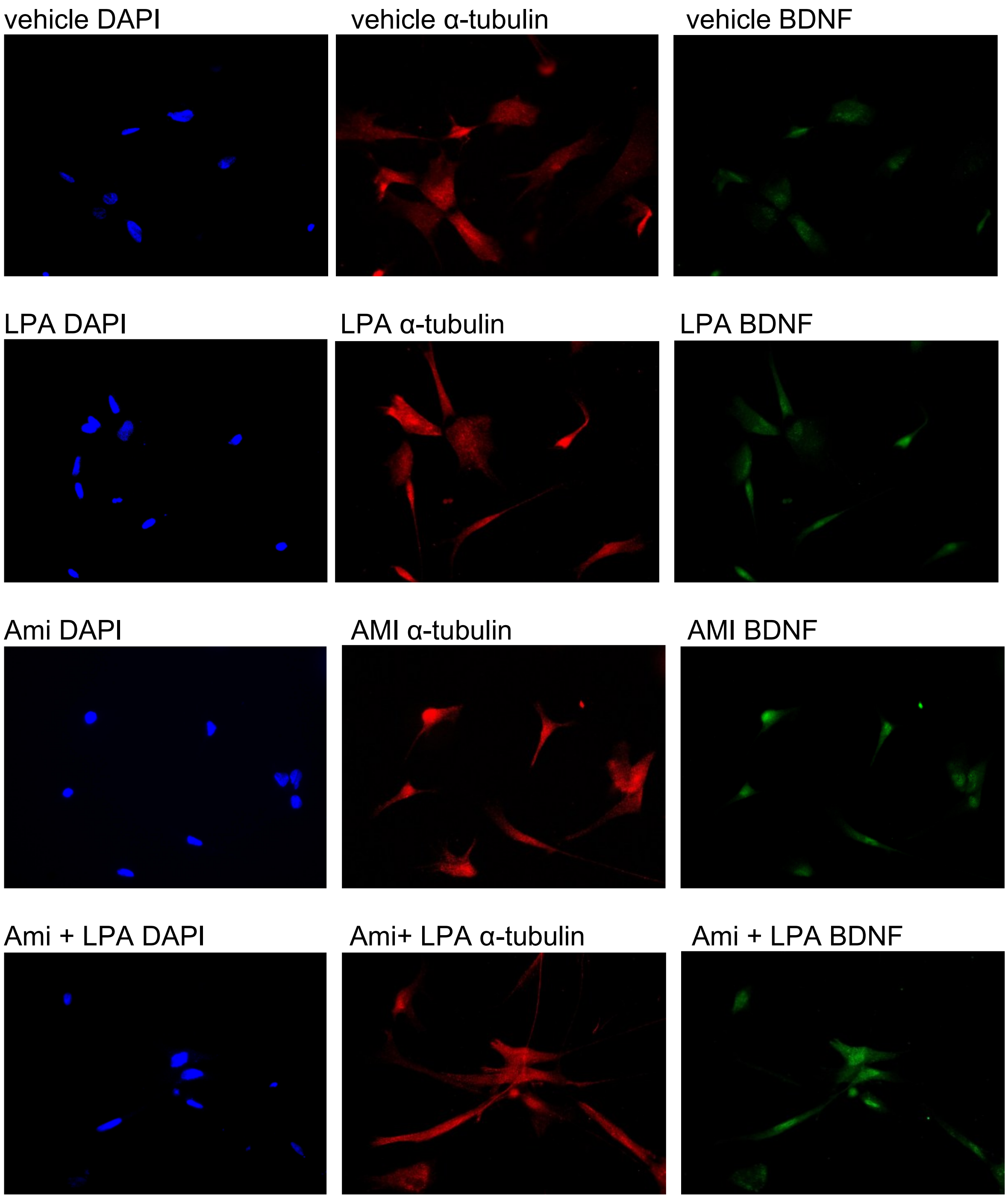
vehicle DAPI
vehicle α-tubulin
vehicle BDNF
LPA DAPI
LPA α-tubulin
LPA BDNF
Ami DAPI
AMI α-tubulin
AMI BDNF
Ami + LPA DAPI
Ami+ LPA α-tubulin
Ami + LPA BDNF

Figure 3C

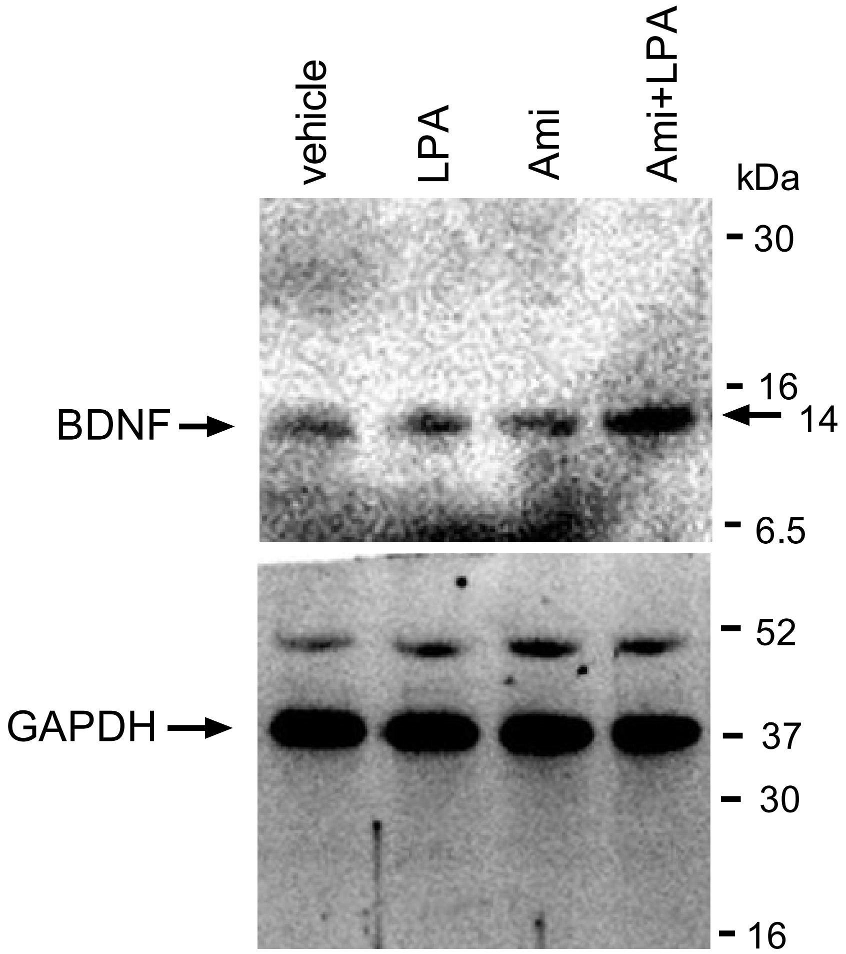

vehicle
LPA
Ami
Ami+LPA
kDa
30
16
14
6.5
BDNF
52
37
30
16
GAPDH

Figure 4A

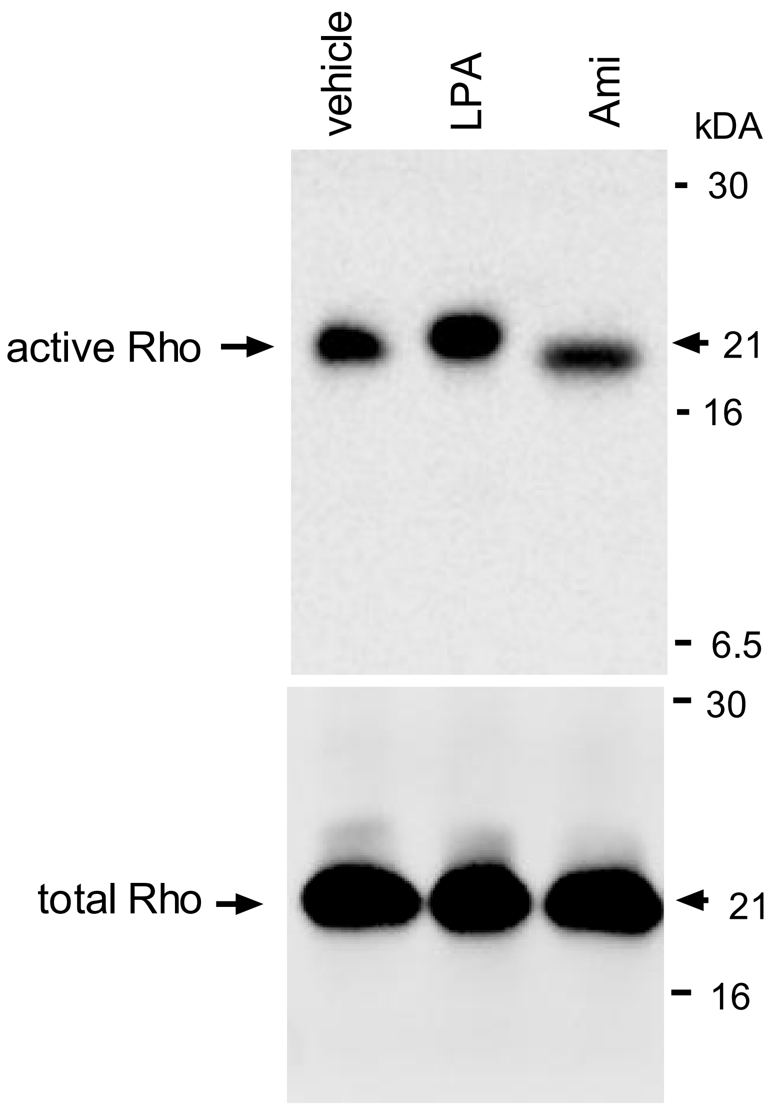

Figure 4B

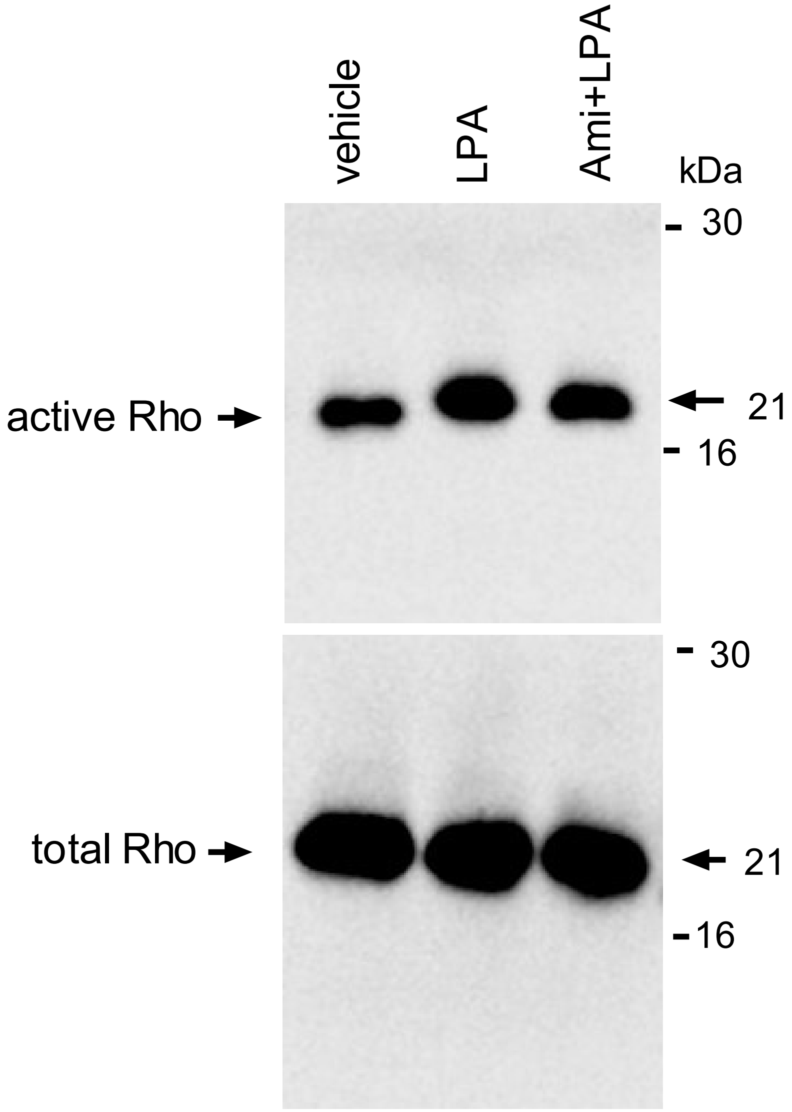
vehicle
LPA
Ami+LPA
kDa
30
active Rho
21
16
30
total Rho
21
16

Figure 5

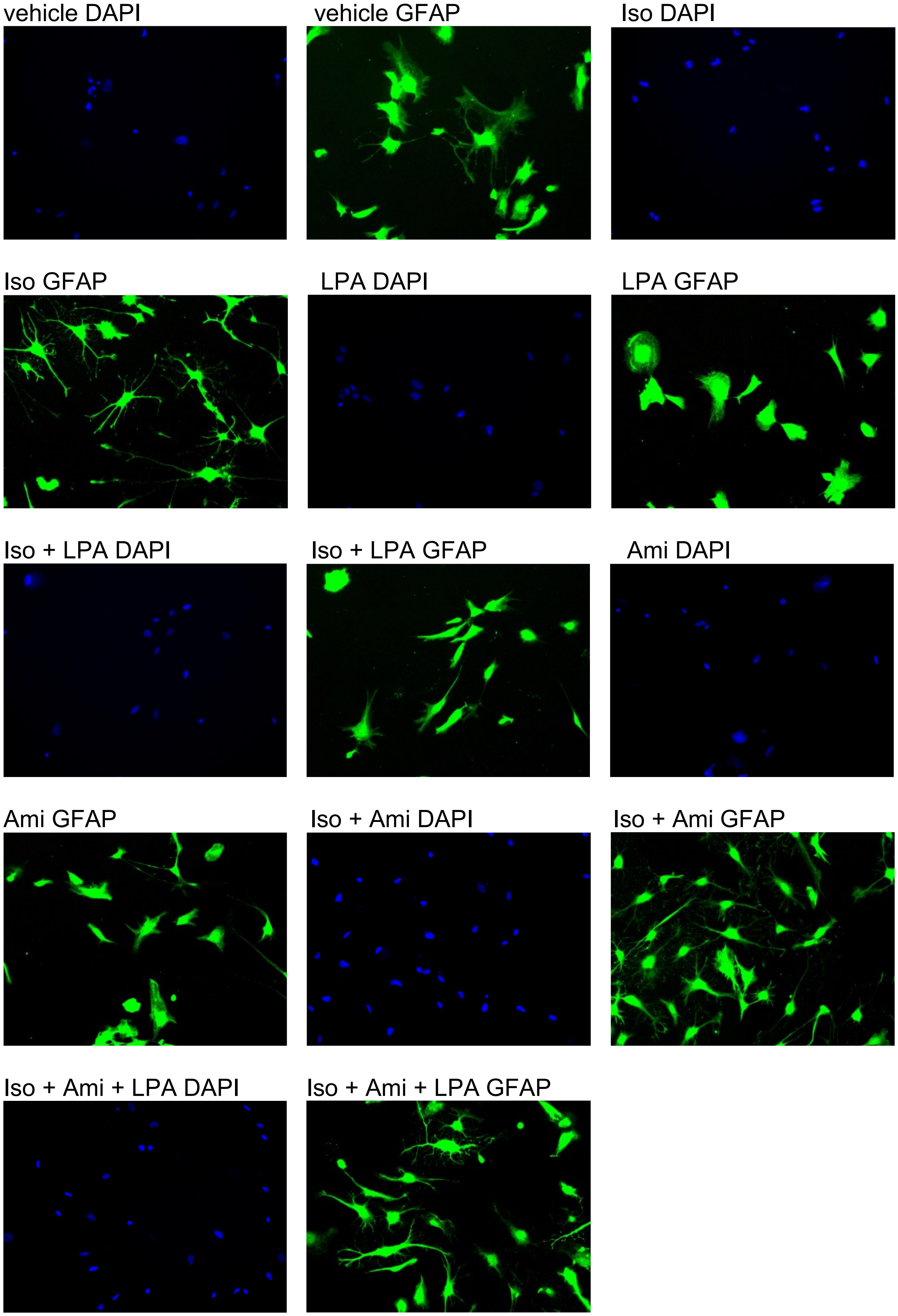
vehicle DAPI
vehicle GFAP
Iso DAPI
Iso GFAP
LPA DAPI
LPA GFAP
Iso + LPA DAPI
Iso + LPA GFAP
Ami DAPI
Ami GFAP
Iso + Ami DAPI
Iso + Ami GFAP
Iso + Ami + LPA DAPI
Iso + Ami + LPA GFAP

Figure 6A

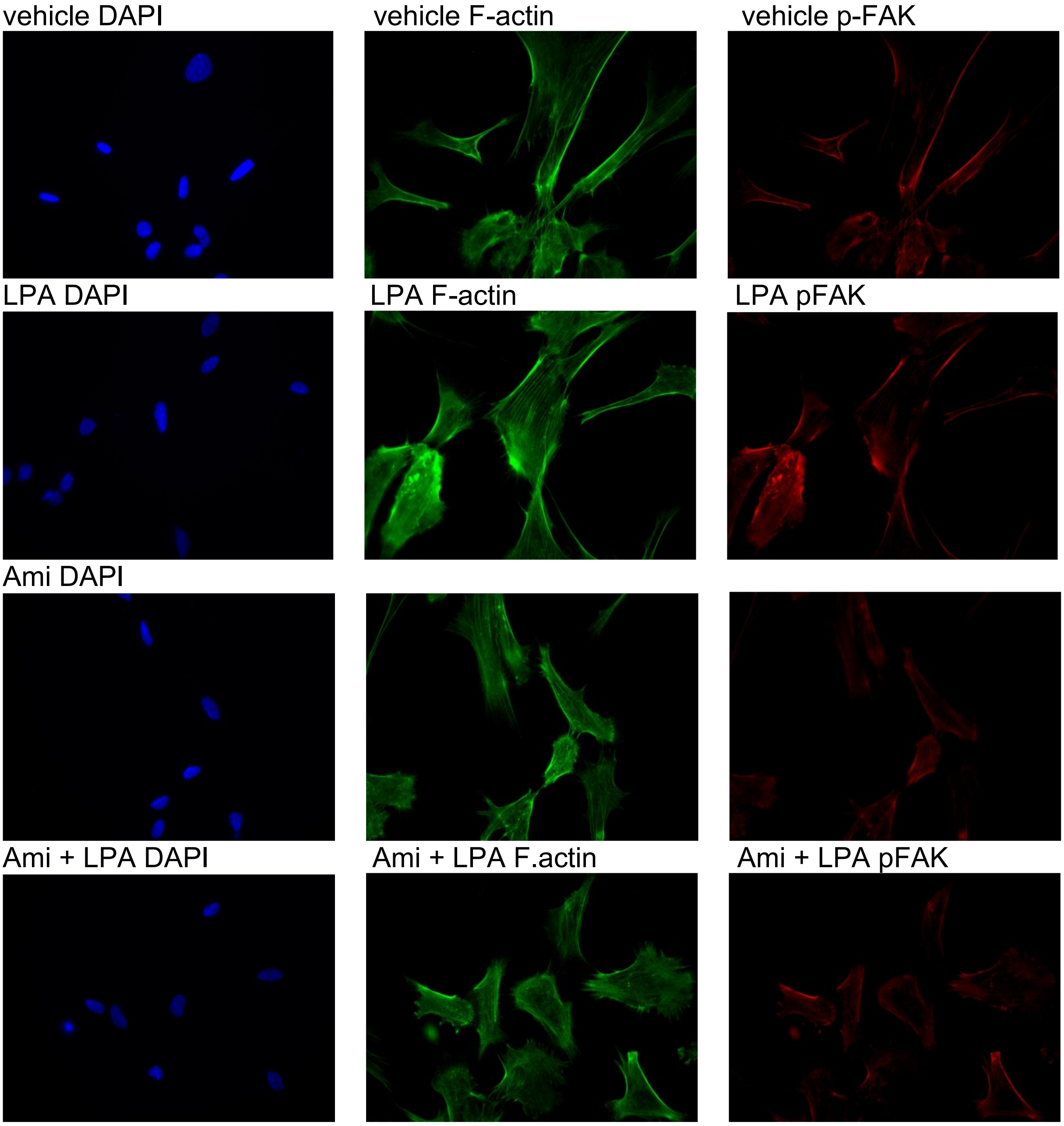

Figure 6D

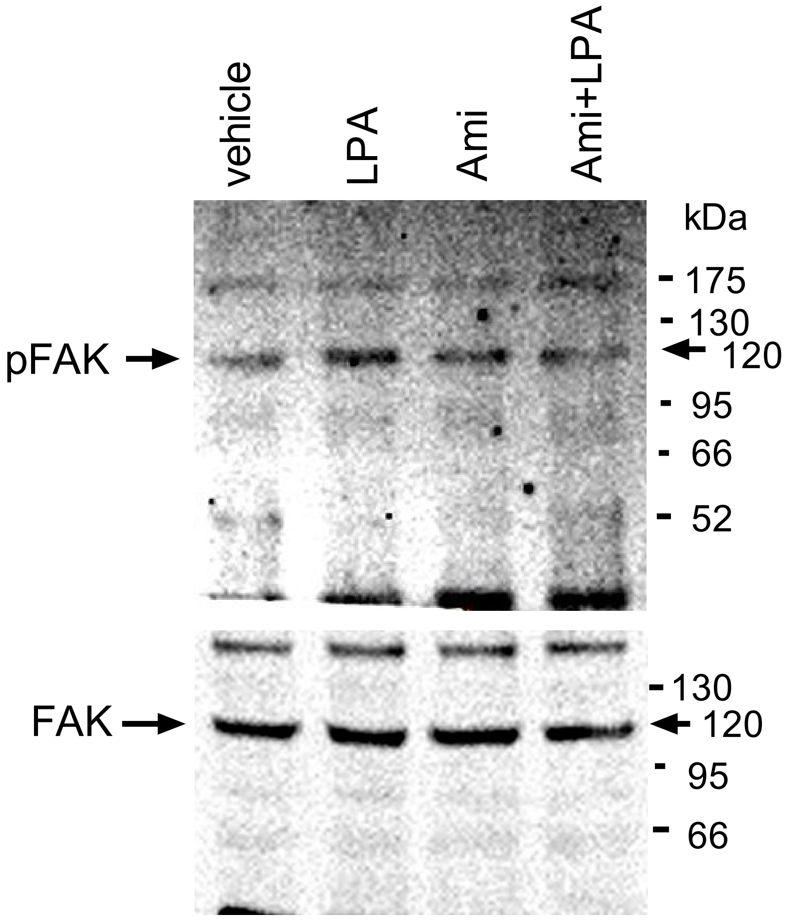
vehicle
LPA
Ami
Ami+LPA
kDa
175
130
120
95
66
52
pFAK
FAK
130
120
95
66

Figure 7A

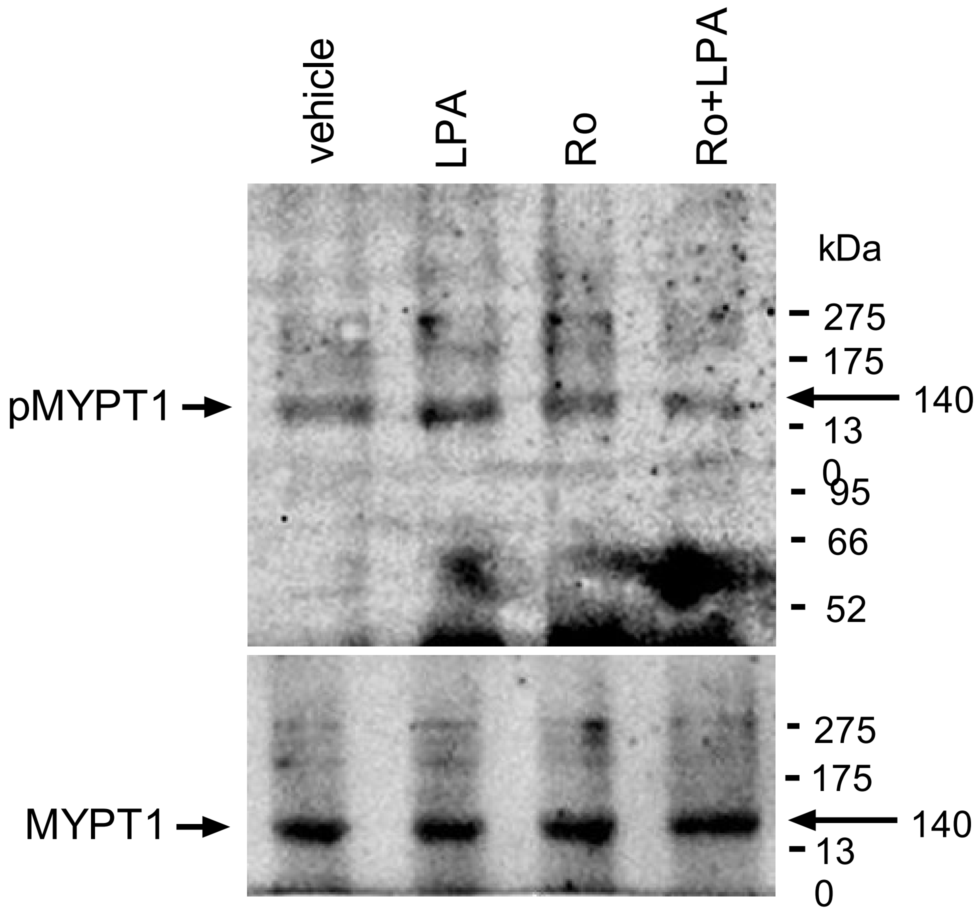

Figure 7B

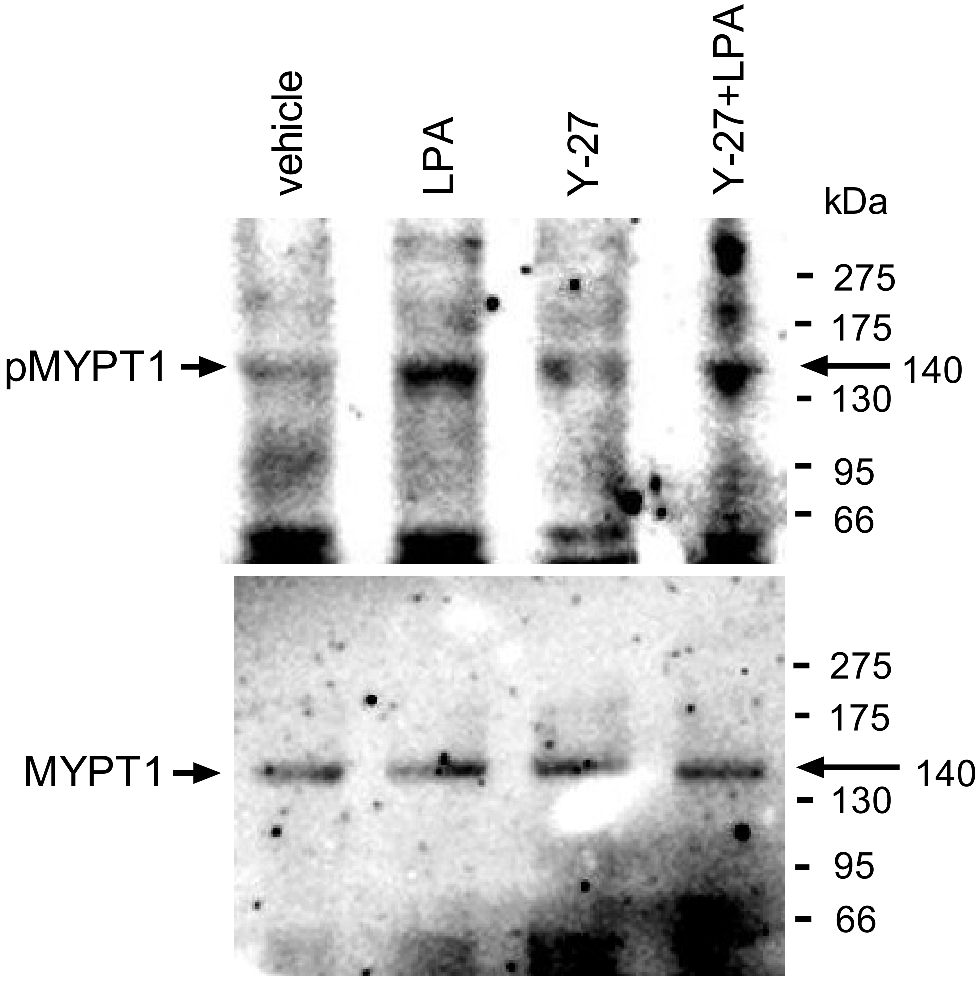
vehicle
LPA
Y-27
Y-27+LPA
kDa
275
175
pMYPT1
140
130
95
66
275
175
MYPT1
140
130
95
66

Figure 7C

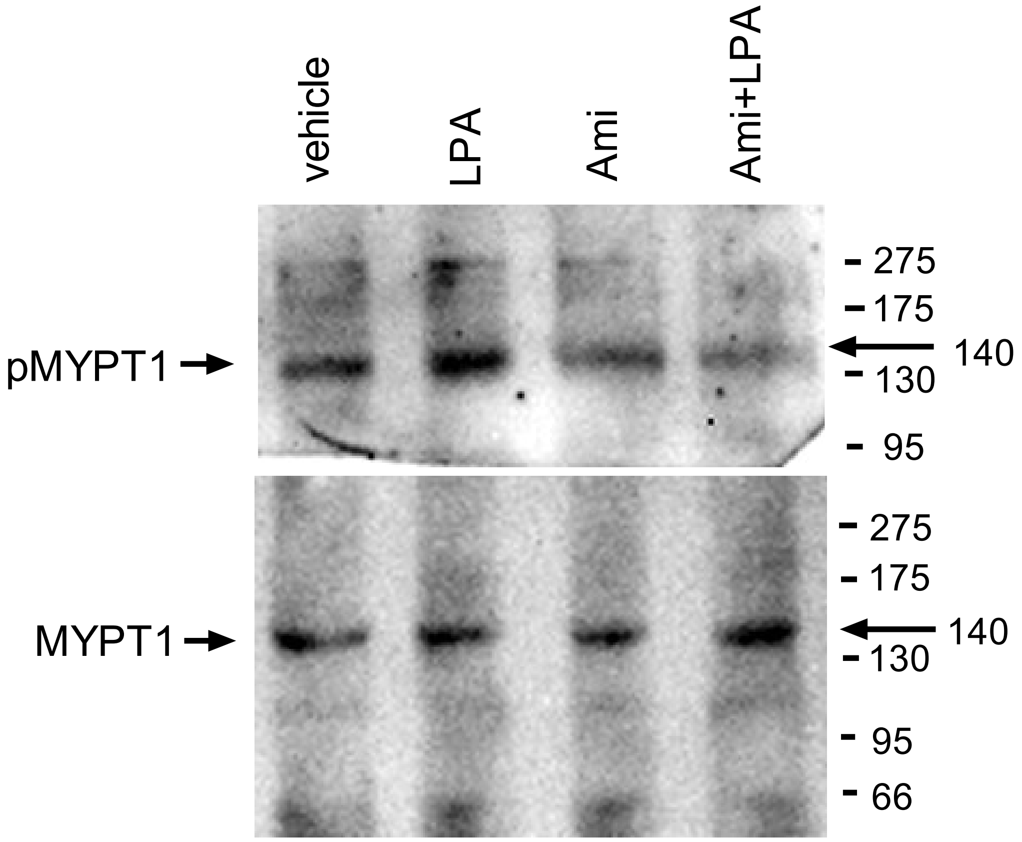

Supplementary Figure 1A

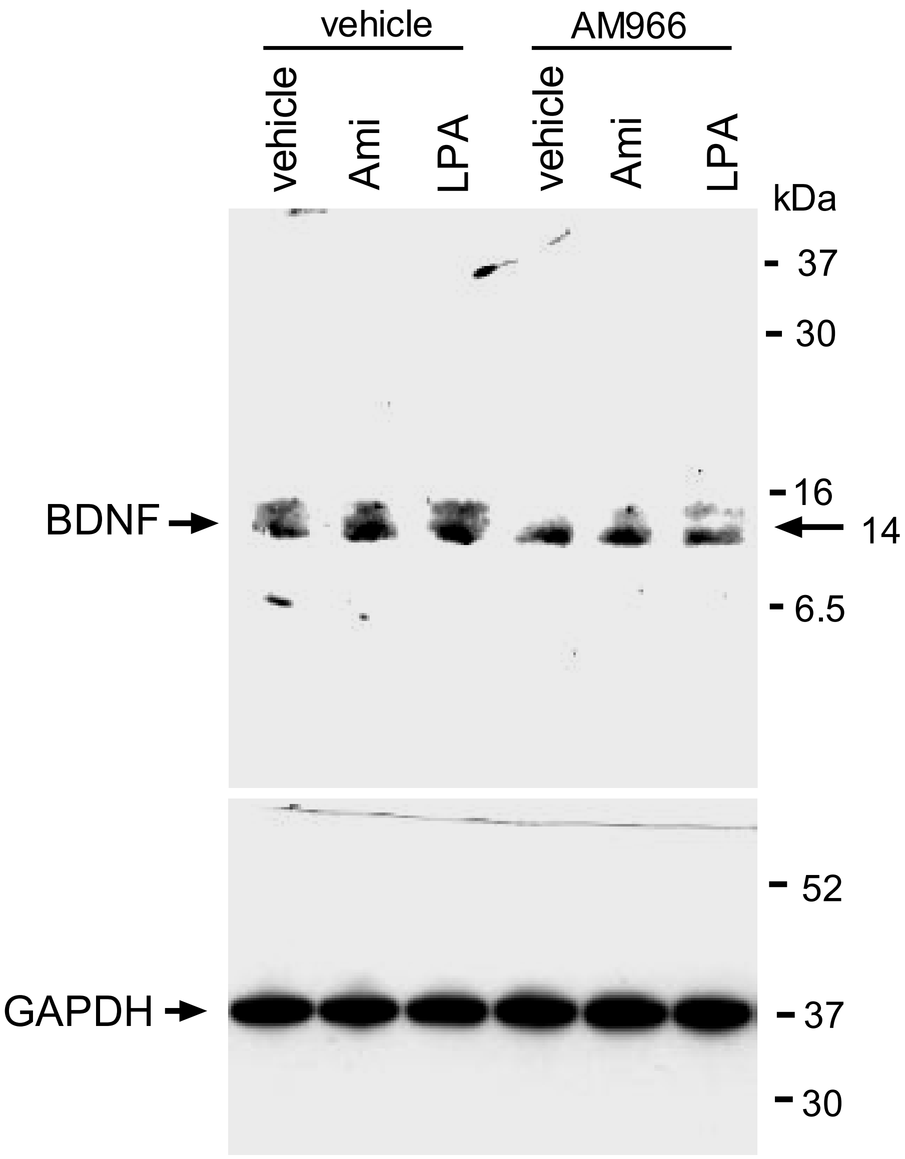
vehicle
AM966
vehicle
Ami
LPA
vehicle
Ami
LPA
kDa
37
30
16
14
6.5
BDNF
52
37
30
GAPDH

Supplementary Figure 1B

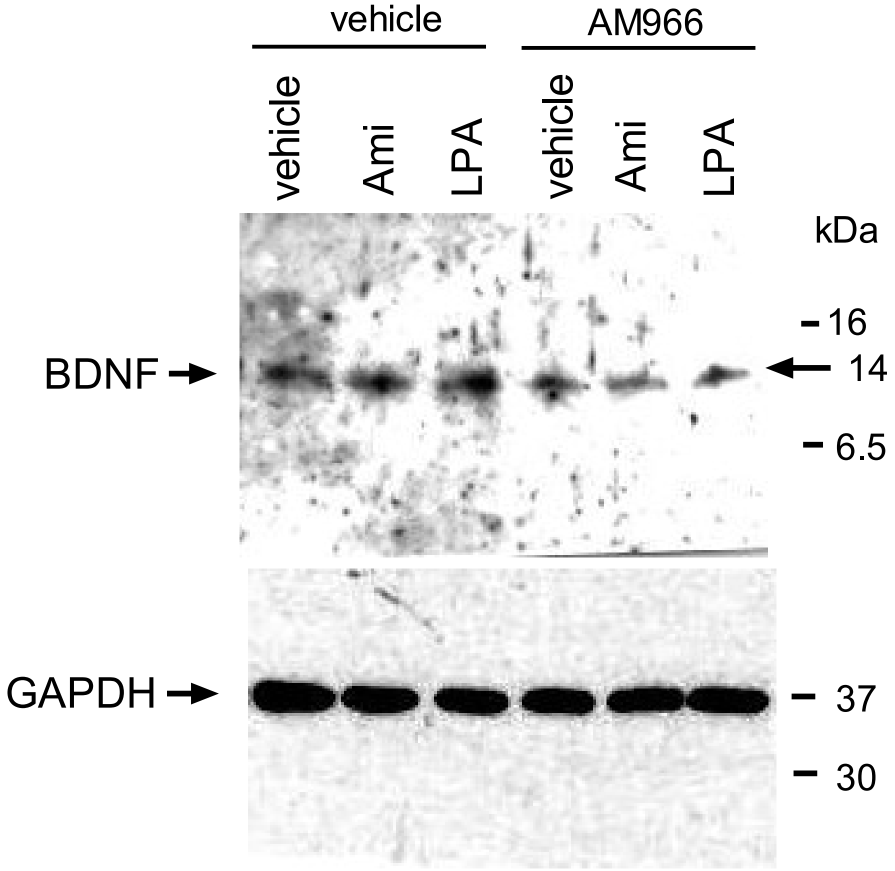

Supplement: Supplementary file 1 [file ijms-27-03660-s001.zip › Figure S1-supplementary information IJMS 2026.pdf]
